# Delivering Phytochemicals to the Implantation Window: The Dual Constraint of Achieving Endometrial Exposure Without Compromising the Embryo

**DOI:** 10.3390/ph19101620

**Published:** 2026-10-12

**Authors:** Yohan Seo, Joohan Woo, Seong Woo Choi

**Affiliations:** Department of Physiology, Dongguk University College of Medicine, 123 Dongdae-ro, Gyeongju 38066, Republic of Korea; ddukdae12@dongguk.ac.kr

**Keywords:** endometrial drug delivery, endometrial receptivity, implantation window, first uterine pass effect, phytochemical bioavailability, embryo safety, nanocarriers, vaginal delivery, decidualization

## Abstract

Whether a phytochemical promotes or suppresses endometrial receptivity is context-dependent, and the dose axis of that context cannot be placed on a concentration scale without knowing how much compound reaches the endometrium—a measurement this review’s documented search did not retrieve for the human endometrium. This review asks why oral administration does not produce interpretable endometrial exposure, which routes can, and what safety constraint follows from targeting an endometrium that contains an embryo. Poor oral bioavailability is not a single obstacle: absorption failure, loss of the parent compound, intestinal conjugation, systemic metabolism and host-dependent speciation overlap, and what differs by class is which step dominates. Endometrial targeting is not unprecedented—vaginal progesterone attains endometrial concentrations disproportionate to serum through the first uterine pass, quantified in human tissue—but that precedent is specific to a small lipophilic steroid and to the released drug rather than the carrier. Uniquely, this target is dual-constrained: a carrier must reach the tissue without exposing an embryo that lipid nanoparticles reach by crossing the zona pellucida. Peri-implantation delivery therefore requires context-dependent design choices: release aligned to a defined window, bounded exposure, and separate verification of carrier distribution, released-drug exposure, endometrial effect and developmental outcome.

## 1. Introduction: The Unmeasured Concentration

A body of evidence indicates that the direction in which a phytochemical shifts endometrial receptivity is not a fixed property of the molecule: the same compounds appear in one literature as promoters of receptivity and in another as suppressors of endometrial proliferation, inhibitors of decidualization, or candidate contraceptives. The resolution of that contradiction turns on context—receptor milieu, dose, timing, tissue state, and the endpoint measured. That context-dependence is not hypothetical. For resveratrol, the same compound inhibits or promotes decidual transformation of human endometrial stromal cells in two studies that differ in several design features, including the phase at which it is applied [1,2,3]. Quercetin enhances decidualization of human endometrial stromal cells through AKT–ERK–p53 signaling at 25 µM [4], and it does so at least in part by clearing senescent decidual cells: quercetin and dasatinib each reduce senescence markers while markedly increasing the decidualization markers *IGFBP1*, *PRL* and *FOXO1* [5]. The same compound therefore acts on decidualization through a senescence-clearing mechanism whose consequence depends on how much senescence is present—which is itself a property of the decidual phase rather than of the molecule.

The dose axis is peculiar among these contextual variables, and the reason is worth stating precisely rather than globally. Receptor context, exposure timing, tissue state and endpoint can each be specified in an experimental design and reported. Dose can be specified too—an administered dose is always known—but the administered dose is separated from the biological response by four further quantities: systemic exposure, total tissue concentration, uterine fluid concentration, and the pharmacologically available (unbound) fraction at the target [6,7]. Only the first of those four has been characterized for these compounds. Systemic exposure is reported for all five classes, and Section 2 is built on it, but it stands in no fixed relation to the tissue, and in the literature retrieved by the search described in Section 1.2, none of the remaining three was reported for any of the five classes at the human endometrium.

What follows from this is narrower than a claim that the dose–response literature cannot be interpreted at all, and the narrower claim is the one defended here. A study that administers a dose and measures an outcome tests that dose against that outcome, and the inference is valid within the system studied. What the missing measurement prevents is the next three steps: placing results obtained in different systems on a common concentration scale; assigning an in vitro concentration–response curve to an achievable in vivo exposure; and attributing a directional reversal between two studies to concentration rather than to the several other respects in which they differ. Differences in study design, in the timing of exposure relative to the decidual program, in the hormonal and senescent state of the tissue, and in endpoint selection are at least as likely to explain conflicting results as concentration is. Section 4.2 examines one such pair in detail. The measurement gap is therefore a limit on synthesis and on translation rather than a blanket invalidation of individual studies.

The question matters because exposure is not hypothetical. Complementary and alternative therapies are used by a substantial proportion of couples presenting for fertility care [8], and dietary supplement use before conception is prevalent enough to be studied as an exposure in its own right in preconception cohorts [9]; the compounds discussed below are therefore being consumed during the interval this review concerns, whether or not they are prescribed.

This review takes that observation as its starting point and asks three questions. Why does oral administration fail to produce an interpretable endometrial exposure? What routes and carriers could produce one? And what safety constraint follows from targeting an endometrium that, during the implantation window, contains an embryo?

The answers are developed as follows. Section 1.1 and Section 1.2 set out the position of this review and its search strategy. Section 2 disaggregates “poor oral bioavailability” into several overlapping failure modes and identifies which dominates in each class. Section 3 sets out the routes that reach the endometrium and shows that endometrial targeting is a quantified, clinically established phenomenon for one steroid, a precedent the retrieved literature has not extended to phytochemicals. Section 4 specifies the dual constraint that distinguishes peri-implantation delivery from most other targeting problems. Section 5 examines what has and has not been measured and sets a minimum standard for reporting exposure. Section 6 translates the analysis into class-specific strategies, and Section 7 states what would have to be built.

The scope is deliberately narrow. This is not a review of phytochemical efficacy in reproduction, nor of nanomedicine in general. It is an argument that a specific measurement gap is a principal obstacle to resolving the efficacy literature and is at the same time a tractable delivery problem.

### 1.1. What Existing Reviews Cover, and What They Do Not

Three adjacent review literatures bear on this question, and the gap lies between them rather than inside any one of them. Reviews of vaginal drug delivery describe the formulation science of the route in detail—mucoadhesive and thermogelling systems, nanocarriers, controlled-release inserts—but take their target indications from infection, contraception and hormone replacement, and do not treat the receptive endometrium as a target with its own requirements [10,11]. Reviews of nanocarrier delivery in pregnancy are oriented to the placenta and to later gestation, where the conceptus is separated from the carrier by a barrier tissue, so the question of direct carrier access to the embryo does not arise in the form it takes here [12,13]. Reviews of phytochemical pharmacokinetics catalog absorption and metabolism thoroughly but stop at the plasma compartment [14,15,16].

The contribution claimed here must be stated narrowly, because receptivity-directed carrier research does exist. Hajipour et al. prepared a sildenafil citrate-loaded, RGD-functionalized nanostructured lipid carrier (92.7 nm, polydispersity index 0.21) and reported that free sildenafil, the unfunctionalized carrier and the RGD-functionalized carrier increased endometrial cell attachment in an in vitro implantation assay by 1.6 ± 0.4, 1.7 ± 0.3 and 2.3 ± 0.3-fold, respectively, with upregulation of *LIF* and *VEGF* [17]; the same group later applied the approach to a phytochemical payload (Section 3.6). These studies establish that targeted carriers have been directed at endometrial receptivity as an endpoint. What they do not supply is a measured endometrial concentration, an in vivo demonstration during a human implantation window, or any assessment of the embryo. The contribution of the present review is therefore not the observation that endometrial carriers are absent—they are not—but the integration of two requirements that the existing work treats separately or not at all: that peri-implantation exposure be measured, and that embryo safety be established as a specification of the carrier rather than inferred from systemic dose.

### 1.2. Scope, Literature Search and Selection of Compound Classes

This is a narrative review built on a structured and documented search, reported as an evidence map rather than as a systematic review with quantitative synthesis. The distinction is deliberate and is not an argument against reproducibility: the absence of a common effect measure makes meta-analysis impossible, but it does not excuse an undocumented search, and the full strategy is therefore reported here and in the Supplementary Material rather than being available on request.

PubMed was searched through the E-utilities interface. The primary search covered records indexed from database inception to 31 May 2026 and was re-executed in full on 2 October 2026 to verify reproducibility and to capture records indexed in the interval. The retrieval counts reported below are those obtained on 2 October 2026 with the date limit ‘(“1900”[PDAT]:“2026/05/31”[PDAT])’ applied, so that every count can be reproduced exactly. Eighteen queries were run in four blocks. Block A (five queries) combined each compound class with pharmacokinetic terms. Block B (six queries) combined the target and the delivery problem—endometrium, endometrial receptivity and the implantation window, decidualization, the vaginal and intrauterine routes, preimplantation embryo exposure to particles, and human endometrial and embryo tissue models. Block C (six queries) addressed the review’s central negative claim directly, combining each compound class with endometrial terms and concentration terms. Block D was a single methodological control, described below. Queries were restricted to title and abstract fields, with truncated terms given unquoted; no language or article-type filter was applied at the query stage, although only English-language records were ultimately included, which is a limitation acknowledged in Section 8. The complete query strings, their retrieval counts and the screening outcome are given in Supplementary Table S1.

Blocks A and B are scoping searches and were screened by title and abstract to a retrieval ceiling of 500 records per query, yielding 4066 unique records after deduplication. Block C is the evidentiary basis of the negative claim and was therefore screened in full: 115 records were retrieved (curcuminoids 13; carotenoids 0; flavonoids 23; stilbenes 18; phytoestrogens 61; an explicit tissue-concentration phrase across all five classes 0), of which 105 remained after deduplication. Every one of the 105 abstracts was retrieved in full and screened against a single question: does this record report a concentration of one of the five compound classes measured in reproductive tissue or in uterine fluid? Screening was performed in two passes. The first applied a text search across all 105 abstracts for co-occurrence of a tissue term, a concentration term and one of the compound names, which flagged 11 records. The second examined those 11 together with a further 19 whose titles indicated in vivo administration with a reproductive-tissue endpoint, or an analytical method applied to tissue, giving 30 records assessed sentence by sentence. For every concentration reported in any of the 105, the matrix in which it was measured was recorded. Screening used complete abstracts rather than full texts, on the reasoning that a study measuring a compound in reproductive tissue would state as much in its abstract; the residual risk—a tissue concentration reported only in a results table of a paper whose abstract does not mention it—is acknowledged in Section 8 rather than excluded. Supplementary Table S2 lists the outcome, the matrix and the reason for every record.

One record met the criterion, and it qualifies the review’s central claim rather than overturning it. Eason et al. report uterine tissue and serum concentrations of total genistein in young adult rats exposed to dietary genistein or soy protein isolate from conception, with tissue concentrations higher in the genistein-fed group [18]. This is a measured concentration of a compound in one of the five classes in uterine tissue, and it is acknowledged as such throughout. Its distance from the quantity this review requires is nonetheless substantial: it is rat rather than human, uses the whole uterus rather than the endometrium, is total (conjugated plus free) rather than speciated, and follows lifetime dietary exposure rather than a defined dose given during a receptive interval. The claim defended below is therefore the precise one—that no retrieved study reports an endometrial tissue or uterine fluid concentration of these compounds in women—and not the broader claim that no uterine measurement of any kind exists.

A second record is informative for the opposite reason. Kodarahmian et al. randomized 34 women with endometriosis to oral resveratrol 400 mg or placebo for 12–14 weeks and sampled endometrial tissue, endometrial fluid and blood before and after the intervention; the analytes measured were MMP-2 and MMP-9, and resveratrol itself was not quantified in any compartment [19]. The sampling this review calls for was therefore performed in women taking a phytochemical, but the compound was not measured. The three remaining near-misses were an in vivo murine study of EGCG reporting endometrial *VEGF* protein and miR-16-5p expression with no EGCG measurement [20]; a human endometrial xenograft study reporting receptor and proliferation endpoints after resveratrol pellets of defined mass with no tissue assay [21]; and a cohort study reporting urinary, not endometrial, phytoestrogen concentrations [22].

A negative finding of this kind is informative only if the query form is capable of returning a positive one, so Block D applied the same concentration terms to compounds for which endometrial tissue measurements are known to exist. That control query (progesterone, estradiol or danazol, with endometrial and tissue-concentration terms) returned 102 records and retrieved the human endometrial tissue measurements discussed in Section 3.3, including the ex vivo perfusion data of Bulletti et al. [23], the randomized comparison of Ficicioglu et al. [24] and the summary of de Ziegler et al. [25]. The search strategy therefore retrieves endometrial tissue-concentration studies when they exist, and the null result in Block C is a property of the literature rather than of the query. The same control also provides an internal check on citation accuracy: a vaginal-tablet pharmacokinetic study frequently cited in this area [26] was *not* retrieved by the control query, consistent with its reporting only plasma pharmacokinetics—a point taken up in Section 3.3.

Reference lists of retrieved reviews were examined for additional primary sources. Every record cited was retrieved from PubMed, and its bibliographic fields were verified against the PubMed record.

The five compound classes were selected on two criteria: each is among those most frequently proposed or consumed for endometrial or fertility indications, and each has a published human or in vivo pharmacokinetic characterization sufficient to locate its point of failure. Classes lacking the latter—lignans, anthocyanins and most alkaloids among them—are excluded not because they are irrelevant but because the argument of this review requires a known pharmacokinetic failure point, and for those classes it has not been established.

The negative claims made below—that endometrial tissue or uterine fluid concentrations have not been reported for these compounds in women, with the single rat uterine measurement noted above as the nearest exception—refer to what this search retrieved. They are claims about a body of literature that was searched, not proofs of non-existence, and are stated in that form throughout.

## 2. Oral Bioavailability Fails at Several Points, and the Dominant Point Differs by Class

“Poor oral bioavailability” is used across the phytochemical literature as though it names a single obstacle with a single class of solution. It does not. In the five compound classes most often proposed for endometrial support, the route from ingestion to tissue can be interrupted at five mechanistically distinct points: absorption, chemical identity of the absorbed species, intestinal conjugation, systemic metabolism, and host-dependent microbial speciation.

Three qualifications govern the sections that follow, and they are stated here because the classification is easy to over-read. First, these barriers are not mutually exclusive, and none belongs to a single class. Every compound considered here encounters more than one: curcumin is limited both by solubility and by conjugation; resveratrol is conjugated in the intestine as well as cleared systemically; flavonoid aglycones are conjugated intestinally and then also subject to hepatic and renal elimination. What follows identifies, for each class, the step that the available pharmacokinetic evidence indicates is rate-limiting—the step whose relief would change the delivered exposure most—not a step that operates there and nowhere else. Second, the evidence base for each assignment differs in species and in strength, and this is recorded explicitly, class by class, in Section 6 rather than left implicit. Third, several assignments rest on a single compound. The crocin data are from rats and concern crocin specifically; the equol metabotype data concern daidzein specifically. Neither should be extended to the carotenoid or phytoestrogen class as a whole, and the sections below are worded to avoid doing so.

### 2.1. Absorption as the Dominant Limitation: The Curcuminoids

Curcumin is the limiting case. In an open-label phase 1 study, ten healthy male volunteers received a single 12 g oral dose, and plasma curcumin was not detectable [27]. The analytical limit matters to the interpretation and is therefore stated: the lower limit of detection of the assay used was 1 µg/mL, so the result establishes that plasma curcumin remained below 1 µg/mL after 12 g, not that absorption failed completely. Within that bound, the direction is unambiguous—a 12 g dose producing less than 1 µg/mL is a very large discrepancy between dose and systemic exposure—but the claim defended here is the bounded one.

The mechanism is also not purely absorptive. Low aqueous solubility, chemical instability at intestinal pH and rapid intestinal and hepatic conjugation have been identified as the joint cause [14], and co-administration of piperine, which inhibits glucuronidation, produces a substantial increase in systemic availability in human volunteers [28]. This establishes that conjugation contributes materially, so that curcumin exemplifies the overlap described above rather than a pure absorption defect. What is specific to the curcuminoids is the size of the gap between the administered dose and any systemic measurement of it; whether relieving conjugation alone would close that gap has not been established, and the piperine result bounds rather than settles the question.

The formulation response has been extensive. Nanoformulation approaches to curcumin—liposomes, polymeric micelles, solid lipid nanoparticles, phospholipid complexes—constitute a mature literature aimed at raising plasma exposure [29]. No study retrieved in this search reported an endometrial concentration for any of these formulations. The strategies are validated against systemic availability, which is precisely the quantity that does not answer the receptivity question.

### 2.2. Loss of the Parent Compound as the Dominant Limitation: Crocin

For crocin, the failure is different in kind. Following oral administration in rats, the parent glycoside is not detected in plasma, whereas its metabolite crocetin appears at low concentrations; crocin itself is recovered largely from intestinal contents and feces, and is not absorbed after either single or repeated dosing [30]. Whatever activity has been ascribed to crocin in cell culture is therefore, in vivo, an activity of a different molecule at a different concentration. This characterization is of crocin in the rat, and the generalization it supports is correspondingly narrow: it identifies a failure mode—the administered molecule is not the circulating molecule—and does not establish that other carotenoids behave this way, or that the quantitative pattern transfers to humans. A systematic assessment of saffron preparations in female reproductive disorders reports clinical signals across several indications [31], but these are signals from an exposure whose molecular identity at the tissue is not established.

No amount of absorption enhancement resolves this, because the compound tested and the compound delivered are not the same. The only coherent strategies are to bypass, by a non-oral route, the gastrointestinal step at which the parent compound is lost, or to deliver the absorbed species directly. The latter has clinical precedent outside reproduction: a crocetin derivative, trans sodium crocetinate, has been administered intravenously in humans in an oncology trial [32], establishing that the species is formulable and tolerated.

### 2.3. Intestinal Conjugation as the Dominant Limitation: The Flavonoids

Flavonoids are absorbed, but they do not arrive intact. In a single-pass intestinal perfusion model in mice, naringenin showed high permeability throughout the gastrointestinal tract together with extensive local metabolism, the recovered compound appearing predominantly as sulfate conjugates rather than as the aglycone [33]. The model is murine, and the absolute proportions should not be transferred to humans, but the qualitative finding—absorption without arrival of the parent compound—holds in humans: dietary quercetin and kaempferol circulate predominantly as glucuronides and sulfates [34], and the general finding across the polyphenol literature is that the circulating metabolome differs systematically from the ingested compound [15,16]. Intestinal conjugation is not confined to this class—it is also a major contributor for resveratrol (Section 2.4) and for curcumin (Section 2.1)—but for the flavonoids, it is the step that absorption data identify as rate-limiting because permeability itself is high.

Whether this abolishes activity is not settled, and that uncertainty is itself informative. Endothelial cells have been shown to deconjugate resveratrol metabolites back to the free compound [35], raising the possibility that conjugates act as circulating reservoirs whose local hydrolysis determines tissue exposure. Whether that mechanism operates in the endometrium has not been tested in any retrieved study, and its existence means that plasma conjugate concentration is not a usable proxy for active endometrial concentration in either direction.

### 2.4. First-Pass Metabolism and Systemic Clearance: The Stilbenes

Resveratrol is absorbed efficiently—approximately three-quarters of an oral dose—yet oral bioavailability of the parent compound is below one percent [36,37]. The reason is extensive metabolism in the intestine and the liver, with sulfate conjugation prominent; intestinal conjugation is therefore a major limitation for resveratrol rather than a barrier it escapes [36]. Tissue distribution measurements in rats, made after intravenous dosing to bypass absorption, confirm that the parent compound and its metabolites partition differently across organs [38]. What distinguishes the stilbene case from the flavonoid case is quantitative rather than categorical: absorption is high and the combined first-pass and systemic loss is near-total, which makes the achievable parent-compound exposure the binding constraint. The corresponding intervention is therefore neither an absorption enhancer nor a permeation strategy alone, but control of release kinetics together with metabolic stabilization; methylated analogs with improved bioavailability have been proposed on exactly this basis [36].

### 2.5. Host-Dependent Speciation: Daidzein and Equol

The isoflavone case introduces a variable that formulation cannot touch. The activity most often attributed to daidzein depends on its microbial conversion to equol, and only approximately 30–40% of the adult population is able to perform that transformation following a soy challenge, a capacity attributed in part to diet and to gut microflora composition [39]. Metabotype clustering analyses confirm that host-dependent stratification is a general feature of polyphenol metabolism rather than a peculiarity of isoflavones [40]. The equol finding itself, however, is specific to daidzein; it is not a property of phytoestrogens as a class, and genistein—the other isoflavone most often studied in this setting—is not subject to the same conversion.

The consequence is that two participants receiving an identical dose of daidzein may receive different active species. A trial that does not stratify by metabotype is averaging across two pharmacologies. The only intervention that removes the variable is delivery of the active species itself.

### 2.6. Why the Existing Formulation Literature Does Not Answer the Question

Across all five classes, the formulation literature is large and the endpoint is uniform: plasma concentration. Enhancement strategies have been benchmarked against systemic exposure for curcumin [14,29], for catechins [41], and for polyphenols generally [15]. None of the programs retrieved reports an endometrial tissue concentration.

This is the central diagnosis of the present review, and its force is specific. The existing pharmacokinetic literature measures a compartment that is not the target and that, as Section 3 shows, stands in no fixed relation to it. The ratio between plasma and endometrium depends on the route, and for one steroid, the vaginal route inverts it. A plasma-benchmarked formulation improvement therefore cannot be assumed to produce a proportional improvement at the endometrium, and a plasma concentration cannot be used to decide whether an in vitro effective concentration was achieved there.

## 3. Routes of Access to the Endometrium

### 3.1. Anatomical and Physiological Barriers

The endometrium sits behind a cervicovaginal mucus barrier, a cyclically shed and regenerating epithelium, uterine fluid flux, and a cycle-dependent blood supply. The mucus barrier is the first obstacle to the vaginal route, and it has been characterized quantitatively. Mucus is a size- and interaction-filtering gel: uncoated particles are immobilized by adhesive interactions, whereas dense coating with low-molecular-weight polyethylene glycol (PEG) renders particles effectively non-adhesive and allows transport at a substantial fraction of their rate in water [42,43]. Liposomes surface-coated with polyethylene glycol at approximately 7 mol% diffused through human cervicovaginal mucus only about ten-fold slower than in water, effectively penetrating it, whereas raising PEG content to approximately 12 mol% compromised both agent loading and vaginal distribution—establishing a penetration-versus-loading trade-off as an explicit design variable [44]. Systematic variation in PEG surface density confirms that the window between adhesive immobilization and loading loss is narrow [45], and high-molecular-weight PEG coatings have been shown to produce uniform vaginal distribution in vivo [46].

The opposite strategy—mucoadhesion—is not straightforwardly safer. Mucoadhesive particles can alter the microstructure of the mucus layer and may disrupt its protective function [47]—a side effect that is not acceptable in the peri-implantation setting. Hydrophobic compounds can nonetheless be given mucus-penetrating and mucoadhesive properties through self-emulsifying formulation [48]. The mucus barrier is therefore surmountable, and the engineering trade-offs are known; they have not been optimized for phytochemicals or for this target.

### 3.2. The Design Space of Routes

**Figure 1:**
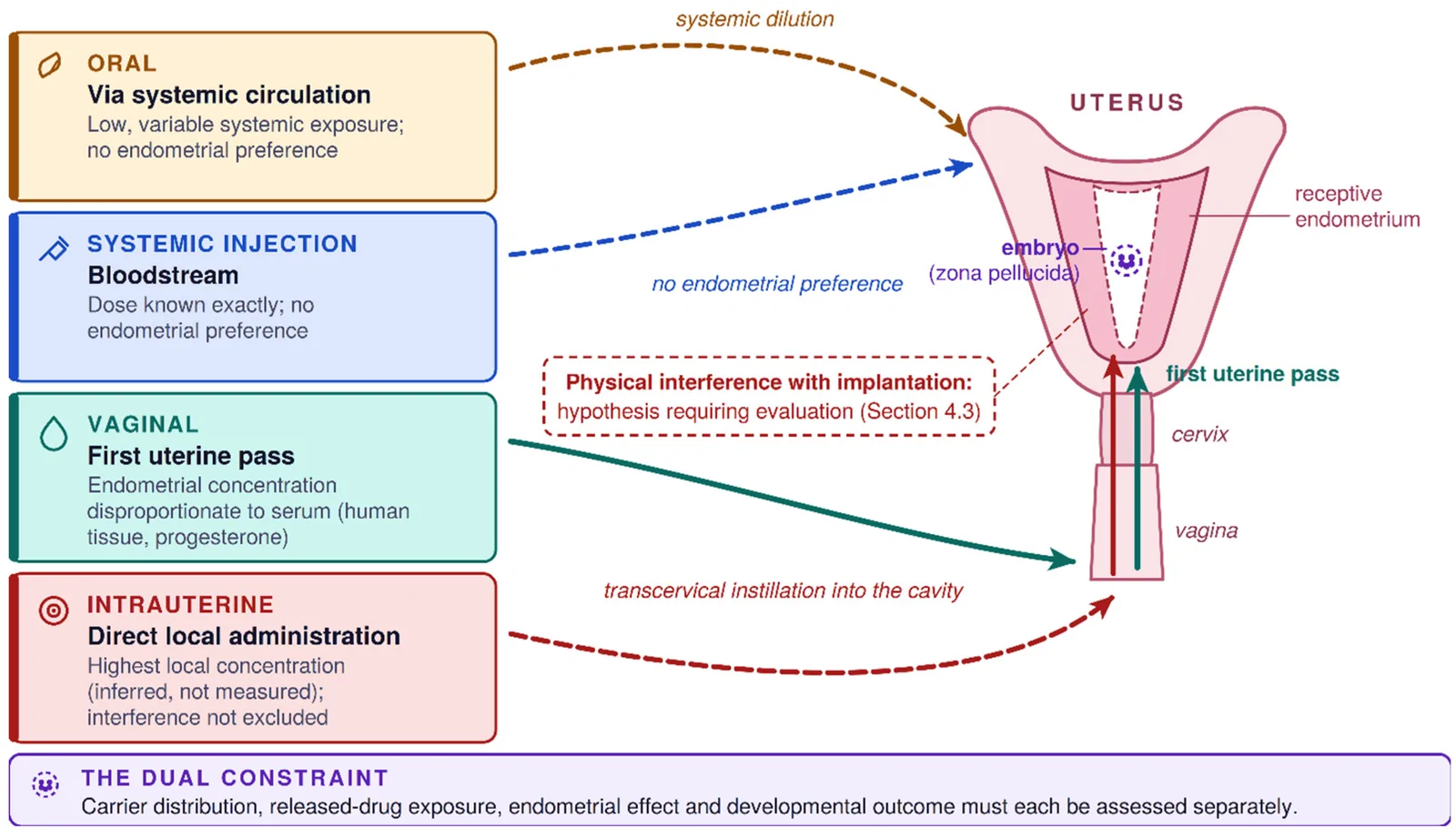
Routes to the endometrium and the dual constraint of the implantation window. Oral, vaginal, intrauterine and systemic routes differ in how they reach the receptive endometrium. The vaginal route achieves endometrial concentrations disproportionate to plasma levels through the first uterine pass, demonstrated in human tissue for progesterone; the intrauterine route gives the highest local concentration but carries a risk of physical interference with implantation that has not been evaluated in a receptive cavity, and is marked here as a hypothesis rather than an established effect. Uniquely, the target contains a second vulnerable participant, the embryo, which lipid nanoparticles can reach by crossing the zona pellucida. The advantage stated for each route is inferred from its pharmacology rather than measured: no head-to-head comparison of endometrial exposure across these routes was retrieved for any phytochemical, and the endometrial concentrations shown for the vaginal route were obtained with progesterone (Table 2). Arrow colour matches the route card: amber, oral; blue, systemic injection; teal, vaginal; dark red, intrauterine. Dashed arrows denote a route whose endometrial delivery is inferred from its pharmacology rather than measured; solid arrows denote the physical path within the reproductive tract, including the first uterine pass demonstrated for vaginal progesterone. The dashed red box and its leader line mark a hypothesis requiring evaluation rather than an established effect. The figure contains no green arrows.

The four routes and the constraint they share are shown in Figure 1 and compared in Table 1. Table 1 records the evidence status of each entry in a separate column, because the four routes are not equally well characterized for this target: the oral and vaginal rows rest on direct human pharmacokinetic data, the systemic row rests on general pharmacological principle, and the intrauterine row rests on a risk that has been neither demonstrated nor excluded (Section 4.3).

**Table 1:** Routes of access to the endometrium. The final column records the kind of evidence on which each row rests. The ordering of routes by achievable local concentration is inferred from their pharmacology and has not been measured for any phytochemical; the intrauterine entry is a hypothesis requiring evaluation (Section 4.3).

Route	Mechanism of Endometrial Access	Advantage	Constraint	Evidence Status	Ref.
Oral	Via systemic circulation	Non-invasive, acceptable	Low and variable systemic exposure; no endometrial preference (Section 2)	Direct human or in vivo pharmacokinetic data for all five classes	[14,27,30,33,36,39]
Vaginal	First uterine pass (direct vagina-to-uterus transfer)	Bypasses hepatic first pass; uterine-preferential distribution	Mucus barrier; expulsion; penetration-versus-loading trade-off	Direct human tissue measurement, for progesterone only (Table 2); not demonstrated for any phytochemical or for an intact carrier	[23,24,25,44,47]
Intrauterine	Direct local administration	Highest local concentration; release over a defined interval feasible	Invasive; physical interference with implantation not excluded—hypothesis, Section 4.3	No study in a receptive cavity with an embryo present; all retrieved systems are deployed in a damaged or non-receptive uterus	[49,50,51]
Systemic injection	Bloodstream	Dose delivered is known exactly; plasma kinetics well defined	No endometrial preference; whole-body and embryo exposure	General pharmacological principle; particle-dependent transfer characterized at the maternal–fetal interface in later gestation only	[52,53]

**Table 2:** Human endometrial exposure after vaginal and systemic progesterone: the primary sources, their conditions and their limits. Values are reported as given in the source; dashes indicate items that do not apply, and “not stated in the published report” marks a value the published report did not make available, as distinct from a quantity the study did not measure, which is marked as such. Bold marks the three entries most often misreported in secondary citation of these studies: the postmenopausal status of the participants, the direction of the serum finding, and the absence of any tissue measurement in the fourth source.

Study	Design and Model	*n*	Formulation, Dose and Route	Hormonal Status	Sampling	Endometrial Tissue Result	Serum Result	Principal Limitation
[23]	Ex vivo perfused human uterus after hysterectomy	Not reported	^3^H- and unlabeled progesterone applied to the vaginal cuff	Uteri obtained in luteal or proliferative phase	4 h (steady state 4–5 h; perfusion to 12 h)	185 ± 155 ng/100 mg endometrium (myometrium 254 ± 305); luteal 280 ± 156 vs. proliferative 74 ± 28 ng/100 mg	Not applicable	Ex vivo; no intact systemic compartment; no embryo; sample size not reported
[24]	Prospective randomized comparison	32	Vaginal progesterone gel vs. intramuscular progesterone in oil, 50 mg	**Postmenopausal**; transdermal estradiol 50 µg/day for 2 weeks	Single timepoint after progesterone	Higher in the vaginal arm (absolute values not stated in the published report)	**Higher in the intramuscular arm**	Postmenopausal, hormone-replaced; not a conception cycle; single timepoint
[25]	Narrative summary of the first uterine pass	—	Vaginal vs. systemic progesterone; also danazol	—	—	Uterine tissue > 10-fold higher after vaginal than after systemic administration	Plasma > 7-fold higher after systemic administration	Summary rather than a primary dataset; per-subject values not given
[26]	Single-dose human pharmacokinetics	Not reported	Vaginal tablet 100 mg vs. gelatin capsule 100 mg	Assisted reproduction; estrogen priming discussed	Serial over 24 h	**Not measured**	C_max_ 31.53 ± 9.15 nmol/L; T_max_ 6.92 ± 3.12 h; t½ 16.39 ± 5.25 h	Plasma only; sometimes cited as tissue evidence, which it is not

### 3.3. The Vaginal Route Is Quantified—For One Molecule

The premise that endometrial exposure cannot be quantified is false, and progesterone establishes this in human tissue. Vaginal administration produces endometrial concentrations disproportionate to plasma levels, a phenomenon termed the first uterine pass effect and attributed to direct vagina-to-uterus transfer rather than to systemic recirculation [23,25]. The primary measurements, their conditions and their limitations are set out in Table 2 rather than summarized in prose, because the differences between them bear directly on how far the precedent extends. Three points require correction or qualification relative to the way these data are commonly cited.

First, only two of the commonly cited sources contain an endometrial tissue measurement, and both are qualified. Bulletti et al. applied tritiated and unlabeled progesterone to the vaginal cuff of an ex vivo perfused human uterus and measured 185 ± 155 ng per 100 mg of endometrial tissue at 4 h, with a steady state reached by 4–5 h; endometrial extraction was higher in uteri obtained in the luteal phase (280 ± 156 ng/100 mg) than in the proliferative phase (74 ± 28 ng/100 mg) [23]. This is a human tissue measurement with preserved architecture, but it is ex vivo, has no intact systemic compartment, and contains no embryo. Ficicioglu et al. randomized 32 postmenopausal women, all receiving transdermal estradiol 50 µg/day for two weeks, to vaginal progesterone gel or intramuscular progesterone in oil 50 mg, and measured both serum and endometrial tissue progesterone [24]. This is a randomized human comparison, but the participants were postmenopausal and the cycle hormone-replaced rather than a conception cycle, which should be stated whenever the result is applied to implantation.

Second, the direction of the serum finding in that study is the opposite of what is sometimes reported. Serum progesterone was higher in the intramuscular group; endometrial tissue concentration was higher in the vaginal group [24]. The correct statement is therefore that the vaginal route achieved higher endometrial concentrations despite lower serum concentrations—not at comparable serum levels. The same relationship is described quantitatively by de Ziegler et al., who report that uterine tissue concentration after vaginal administration exceeded that after systemic administration by more than ten-fold, despite plasma levels in the systemic case that were more than seven times higher [25].

Third, one frequently cited source does not report an endometrial concentration at all. The vaginal-tablet study of Levy et al. reports plasma pharmacokinetics after a single 100 mg dose—C_max_ 31.53 ± 9.15 nmol/L, T_max_ 6.92 ± 3.12 h, terminal half-life 16.39 ± 5.25 h, with a higher C_max_ than a gelatin capsule of the same dose—and no tissue measurement [26]. It is cited in this review for plasma pharmacokinetics only. The control search of Section 1.2 confirms this independently: a query built on endometrial tissue-concentration terms retrieved Bulletti, Ficicioglu and de Ziegler but did not retrieve Levy.

The clinical corollary of the first uterine pass is well established—vaginal progesterone achieves adequate endometrial effect at serum concentrations far below those required by intramuscular dosing [54,55]—and luteal support by the vaginal route is standard practice in assisted reproduction [56].

Three conclusions follow for the present argument, and a fourth bounds them. First, endometrial tissue concentration is a measurable quantity in humans, so the absence of such measurements for phytochemicals is a gap in practice rather than a limit of method. Second, the same administration yields different tissue concentrations by cycle phase [23], so the dose and timing axes of phytochemical action are coupled at the level of tissue exposure—a coupling not characterized for any phytochemical in the retrieved literature. Third, the first uterine pass is a quantified phenomenon rather than a concept.

The fourth point is the bound, and it is the principal qualification this review places on its own argument. Progesterone is a small, uncharged, highly lipophilic steroid, and the first uterine pass has been demonstrated for it and for danazol [25]—molecules of broadly similar physicochemistry. Nothing in these data establishes that a curcuminoid, a glycosylated carotenoid, a flavonoid conjugate or an isoflavone will partition in the same way, still less that an assembled nanocarrier will. Two distinct transport problems are involved and should not be conflated: transport of a released small molecule across the vaginal and uterine walls, which is what these studies measure, and transport of an intact particle, which they do not address. The progesterone precedent therefore establishes that preferential endometrial exposure is physiologically real and analytically measurable; it does not predict its magnitude, or its existence, for any other molecule or for any carrier. Each would have to be measured.

### 3.4. Vaginal Formulation Platforms Already Exist

The vaginal route is not only quantified but engineered. Mucoadhesive and thermogelling systems, nanocarrier formulations and controlled-release inserts constitute an established formulation science [10,11]. Intravaginal rings deliver a range of agents with defined release kinetics over days to weeks, including antiretrovirals [57] and contraceptive steroids [58] in clinical or human tissue evaluation, and combined estradiol and progesterone in a large-animal model [59]; additive manufacturing has recently been applied to progesterone rings specifically [60].

The relevance is limited in one important respect, developed in Section 4.2: most of this platform science optimizes for release over weeks, whereas the interval of interest here is measured in days. The components are available; the release duration they are tuned to deliver is longer than the one required, a mismatch to be addressed by re-specifying a device rather than by inventing one.

### 3.5. Two Qualifications to the Vaginal Route

Two variables temper the case made above, and both are tractable rather than disqualifying.

The first is the local microbiota. The vaginal route places a formulation in a metabolically active compartment: vaginal bacteria modify the efficacy of topical tenofovir in women, with non-*Lactobacillus*-dominant communities depleting the active drug, and the vaginal microbiome measurably alters topical antiretroviral pharmacokinetics [61,62]. A phytochemical delivered vaginally is therefore subject to a second speciation variable analogous to the gut metabotype problem of Section 2.5, operating this time at the site of administration. No study retrieved in this search has characterized vaginal microbial metabolism of any phytochemical, and the reporting standard proposed in Section 7.1 should accommodate it.

The second is species. The first uterine pass was characterized in women, and the quantitative human data cited in Section 3.3 come from an ex vivo human uterus and from postmenopausal women [23,24]; no equivalent characterization in a common laboratory species was retrieved. Formulation screening in animals is therefore appropriate, but the endometrial-to-plasma ratio obtained in an animal model should not be assumed to transfer, and this review makes no claim about its magnitude outside humans.

### 3.6. Targeted Carriers Exist at an Early Stage and Have Not Faced the Implantation Window

Surface-functionalized nanoparticles for uterine and placental targeting have been developed, and the stage of that development should be stated precisely rather than summarized as maturity: placenta-specific delivery using ligand-decorated nanoparticles reduces maternal off-target exposure in pregnancy complications [12], and placenta-targeted peptide-liposomes, decorated nanoparticles and ultrasound-guided site-selective delivery have been described for gene therapy of the placenta [13]. Trophoblast-targeted liposomes have been formalized as a method [63], and placenta-tropic lipid nanoparticles delivering mRNA have been shown to ameliorate murine preeclampsia [64]. Receptor-directed targeting of the uterus itself has also been attempted: oxytocin receptor-targeted PEGylated immunoliposomes have been synthesized and characterized in vitro [65].

The development stage of this literature is preclinical throughout. The efficacy demonstrations are performed in rodents, the human work is performed in vitro, no clinical trial of a uterine- or placenta-targeted nanocarrier was retrieved, and no retrieved study reports a measured payload concentration in human uterine tissue. What is accurate is that the component technologies—ligand functionalization, particle engineering, defined release—are established in other indications and have reached proof of concept in reproductive tissue.

Work directed specifically at endometrial receptivity also exists and is the closest precedent to the proposal made here. Hajipour et al. functionalized a nanostructured lipid carrier with RGD and loaded it with sildenafil citrate, obtaining 92.7 nm particles with a polydispersity index of 0.21, and reported that free sildenafil, the unfunctionalized carrier and the RGD-functionalized carrier increased endometrial cell attachment in an in vitro implantation assay by 1.6 ± 0.4, 1.7 ± 0.3 and 2.3 ± 0.3-fold, respectively, with upregulation of *LIF* and *VEGF* [17]. This is a targeted carrier aimed at receptivity, and it is acknowledged as such. Its limits are precisely the limits this review is concerned with: the payload is a conventional drug rather than a phytochemical, the endpoint is cell attachment rather than implantation, the system is in vitro, no concentration was measured in endometrial tissue, and no embryo was present or assessed.

Every system described above is directed at the placenta, the myometrium or an in vitro endometrial preparation, and none of those retrieved has been evaluated during an implantation window with an embryo present. The correct diagnosis is therefore not that uterine targeting is impossible, but that the tools exist at an early stage of development and have not been aimed at this target under its own constraints. The proposal here is not to invent a technology but to redeploy and re-specify one—structurally the same logic of reassignment developed for the compounds themselves in Section 6.

The biology supports the approach. During early implantation, extracellular vesicles are exchanged between embryo and endometrium and regulate the process [66], and this embryo–maternal vesicular dialogue is now documented across species and stages [67,68]. A natural nanoscale delivery system therefore operates at the embryo–endometrium interface. This is at once the biological rationale for exosome-based carriers and the origin of the dual constraint of Section 4: a carrier placed in the uterine cavity enters the same channel through which the embryo communicates. Extracellular vesicles have in parallel been developed as engineered drug delivery vehicles with defined loading and targeting strategies [69,70,71]. A direct demonstration with a phytochemical payload exists—epigallocatechin gallate loaded into menstrual-blood-derived exosomes (86.2 nm, zeta potential −11.8 mV, encapsulation efficiency 65.18%) enhanced endometrial cell adhesion and upregulated *LIF*, *HOXA10* and integrin β3 more strongly than the free compound [72]—establishing, in a single study, both that formulation changes the endometrial result and that phytochemical delivery to endometrial cells is achievable. Its limits define the gap precisely: it was conducted in vitro, it reported molecular markers of receptivity and an adhesion index rather than an endometrial concentration, and it was not evaluated for effects on an embryo. It and the Hajipour study are compared with the other principal primary sources in Table 3.

## 4. The Dual Constraint of the Implantation Window

The central claim of this review is that delivery to the implantation window differs from conventional delivery to a tumor or an inflamed tissue because a second vulnerable participant—the embryo—occupies the target site and because the cost of failure is asymmetric: an adverse effect is a failed pregnancy. The term dual constraint is used throughout in this specific sense: the delivery system is simultaneously constrained by what the endometrium requires and by what the embryo must not receive. The four subsections that follow are not four independent constraints but the four design consequences of that single duality—one for the carrier (Section 4.1), one for the release profile (Section 4.2), one for the physical form (Section 4.3), and one for the compound itself (Section 4.4). The asymmetry is compounded because the endometrium is not a passive substrate: decidualizing endometrial stromal cells act as biosensors of embryo quality, leading to mounting differential responses that bias implantation toward or away from a given conceptus [73,74]. An intervention that alters the decidual response therefore alters a selection process, not merely a receptive surface.

Before the consequences are drawn, the interval they apply to should be defined, because several of the arguments below depend on it. The window of implantation is the limited interval in which the endometrium is receptive to blastocyst attachment, conventionally placed at approximately days 19–21 of an idealized 28-day cycle, or 6–10 days after the luteinizing hormone surge, and assessed clinically by histological, molecular or transcriptomic criteria whose relative performance has been systematically reviewed [75]. Two delivery settings follow from this and are kept distinct throughout. In natural or stimulated conception, the time of fertilization is not known precisely, so the position of the window must be estimated and any delivery system must cover a wider interval than the window itself. In frozen or fresh embryo transfer the time of transfer is scheduled, so release can be anchored to a known event and the required coverage is correspondingly narrower. The practical consequence is that the two settings call for different release durations—in the second, coverage of the order of days anchored to transfer; in the first, a wider interval spanning the estimated window—rather than for one device being used in both. Where a statement below applies to only one of these settings, it is identified.

### 4.1. Consequence for the Carrier: Carrier Exclusion Is Necessary but Not Sufficient

The preimplantation embryo is demonstrably vulnerable to particulate exposure. Copper oxide nanoparticles impaired preimplantation development, blocking the morula-to-blastocyst transition through disrupted mitophagy, and the effect was reproduced in human blastocysts, with urinary copper concentrations in assisted-reproduction patients inversely associated with clinical outcome [76]. Zinc oxide nanoparticles are toxic to oocyte and early embryo development [77], and polymethylmethacrylate nanoplastics disrupt early embryonic development in mice [78]; the broader literature on micro- and nanoplastic internalization reaches the same conclusion for reproductive and developmental endpoints [79]. On the maternal side, polystyrene nanoplastics and microplastics are taken up by human endometrial stromal cells in a size-dependent manner, with 100 nm and 1 µm particles internalized more readily than 5 µm particles, and with uptake accompanied by morphological change [80].

These are environmental and metal-oxide particles, and the distinction from therapeutic delivery systems should be preserved rather than elided. They are not formulated for controlled release, their surfaces are not engineered for biocompatibility, and the exposures studied are not those of a dosed carrier. What this literature establishes is that the preimplantation embryo and the endometrial stroma are both susceptible to particulate uptake and injury, which makes susceptibility a reasonable prior for an engineered particle; it does not establish that any particular therapeutic carrier is toxic, and no claim of that kind is made here.

Critically, the vulnerability is not merely one of proximity. Lipid nanoparticles traverse the zona pellucida and transfect the preimplantation embryo, with efficiency and cellular target depending on stage of exposure, and with surface composition governing both penetration and toxicity [81]. That the zona is permeable to macromolecules is a long-established property rather than a recent surprise [82].

This reframes the embryo. It is not a bystander that a carrier can be routed around; it is an accessible target. The dual constraint is therefore not a theoretical caution but a demonstrated physical fact; the same finding supplies a design lead, since surface charge and lipid composition are the determinants of penetrance and are also among the most tractable formulation variables.

It does not follow, however, that keeping the carrier out of the embryo is sufficient for safety, and the framework proposed in Section 7.2 is built on that distinction. At least four exposures must be assessed separately, because a system can satisfy one and fail another.

(i) *Carrier distribution.* Where the intact particle goes, including whether it crosses the zona or contacts the trophectoderm after hatching.
(ii) *Released-drug exposure.* A carrier can remain entirely outside the embryo while releasing a compound that diffuses into it. Released small molecules are not subject to the barrier that excludes the particle, so excluding the carrier places no bound on embryonic exposure to the payload, which must be measured on its own terms.
(iii) *Endometrial effect.* The embryo develops in an environment the carrier is intended to modify. A formulation that alters decidual signaling, uterine fluid composition or the biosensor response described above changes the conditions of implantation even if neither carrier nor payload reaches the conceptus [73,74,83].
(iv) *Developmental outcome.* Blastocyst formation, cell allocation and, where the model permits, post-attachment development, assessed against predefined margins rather than against the absence of a statistically significant difference (Section 7.2).

One corollary deserves emphasis because it inverts a familiar heuristic. In systemic delivery, low plasma exposure is a reasonable surrogate for low fetal exposure. In local peri-implantation delivery it is not, because the embryo is inside the compartment into which the dose is placed. A vaginal or intrauterine formulation can therefore combine minimal systemic exposure with maximal embryonic exposure, and low systemic exposure must not be reported as evidence of embryo safety.

The adjacent literature on nanoparticle behavior at the maternal–fetal interface reinforces the need for direct measurement: transplacental transfer is governed by particle attributes rather than uniformly excluded [52,84], and fetotoxicity of nanoparticles has been reviewed as a mechanistically distinct hazard class [53]. That work concerns a barrier tissue in later gestation and does not transfer to the preimplantation setting, where no such barrier exists. What the retrieved literature does not contain is an assay that characterizes carrier distribution and embryo association for the preimplantation embryo. No standard exists.

### 4.2. Consequence for the Release Profile: Alignment, Not Duration

The argument made here is about alignment and must be separated from an argument against sustained release as such, which this review does not make. Two different things are at issue. Controlled or sustained release engineered to cover the window—a device that maintains exposure across the estimated receptive interval and then stops—is not a liability but a plausible solution, and in the natural-conception setting, where the window position is uncertain, it is the more likely one. What is a liability is exposure that persists beyond the interval in which it is intended to act. The problem is therefore misalignment between the release profile and the window, not duration per se, and the design objective is a release profile whose shape matches a defined interval rather than one that lasts as long as possible.

The evidence that misalignment matters comes from a pair of studies with opposing results, and that pair repays examination before its difference is attributed to timing. Resveratrol represses the decidual marker genes *PRL* and *IGFBP1* and abrogates decidual senescence in primary human endometrial stromal cells [2]; an independent study in the same cell type reports enhancement of decidualization [3]. In an attempt to reconcile the two, it has been argued that resveratrol applied during the initial proinflammatory decidual phase—coinciding with the implantation window—inhibits decidual transformation, whereas the same compound given after that phase may promote it by suppressing decidual senescence [1].

That reconciliation is a hypothesis consistent with the data rather than a result established by them, and Table 3 sets out why. The two studies differ in several respects besides the timing of exposure, concentration among them. Ochiai et al. applied 100 µM resveratrol to eight independent primary cultures concurrently with the decidualization stimulus (8-bromo-cAMP and progesterone) for 4 or 8 days, with *PRL*, *IGFBP1*, senescence markers and the *CRABP2*–RAR axis as endpoints [2]. Mestre Citrinovitz et al. used primary and immortalized endometrial stromal cells, a different stimulus (medroxyprogesterone acetate, estradiol and 8-bromo-cAMP), and added resveratrol to cells that had already begun to decidualize—at days 3 and 5 of the program—over a concentration series of 6.25, 12.5, 25 and 50 µM, reporting a dose-dependent increase in *PRL* and *IGFBP1* together with reduced proliferation [3]. The concentration series and the days of addition are not given in the published abstract and are taken here from the extraction table of a systematic review, which is a secondary source and is identified as such [85].

Set against each other, the two studies differ in concentration as well as in timing, and the difference is not marginal: the inhibitory result was obtained at 100 µM, which is twice the highest concentration at which the enhancing result was obtained. Two explanations are therefore available, and they are not mutually exclusive. Under the timing explanation, resveratrol meeting the acute proinflammatory phase inhibits decidual transformation, whereas resveratrol meeting the established program suppresses decidual senescence and promotes it [1]. Under the concentration explanation, the direction of effect reverses somewhere between 50 and 100 µM, which would make this an instance of the biphasic behavior discussed in Section 4.4 rather than of timing at all. Cell source and differentiation stimulus differ as well and could contribute to either. What can be said is that the timing hypothesis, which this review previously treated as the resolution of the conflict, is one of two concrete candidates and is not the demonstrated one. Additionally, the experiment that would separate them is now specifiable, since both axes have known values—a factorial design in a single cell system crossing concentration from below 50 µM to above 100 µM with time of addition from the initiation of decidualization to day 5.

Direct evidence that concentration alone can reverse direction in endometrial tissue exists, and it is worth separating from the timing argument. In an estrogenicity assay in Ishikawa cells, resveratrol acted as an estrogen agonist at low concentrations and as an antagonist at high concentrations when combined with estradiol; in human endometrial xenografts in immunodeficient mice, resveratrol pellets reduced *ESR1* expression and Ki-67 at the highest dose tested (60 mg) [21]. This is an endometrial cancer cell line and a xenograft model rather than a decidualizing primary culture, and the endpoints are proliferative rather than decidual, so it does not settle the decidualization question. It does establish that a concentration-dependent change in direction is not merely postulated for this compound in this tissue.

One further observation belongs here, because it is the only human pregnancy outcome in Table 3 and because its direction matters. In a retrospective cohort of embryo-transfer cycles, continuous oral resveratrol at 200 mg/day was associated with a lower clinical pregnancy rate (odds ratio 0.539, 95% CI 0.341–0.853) and a higher risk of miscarriage (odds ratio 2.602, 95% CI 1.070–6.325) after matching on age, embryo grade and stage, number of embryos transferred, and transfer type [86]. The design is observational and retrospective, the resveratrol group is small relative to the comparison group, supplement composition and adherence were not verified, and confounding by indication cannot be excluded; it is not evidence of causation and is not presented as such. What it does establish is that the concern raised in this section is not purely theoretical. A continuous oral regimen is, by construction, exposure that spans the implantation window rather than one aligned to it, and the one human dataset that examines such a regimen points toward harm rather than benefit. No endometrial concentration was measured in that cohort either, so the association cannot be attributed to a concentration at the tissue—which is the argument of this review restated at the only outcome level that ultimately matters.

The underlying biology explains why temporal sensitivity is plausible. Decidualization proceeds through an acute proinflammatory phase followed by the emergence of stress-resistant and senescent decidual subpopulations, whose balance determines whether the cycling endometrium transitions successfully into the decidua of pregnancy [87,88]. An intervention that suppresses senescence may be beneficial or harmful depending on which phase it meets. Consistent with this, senolytic clearance of senescent decidual cells enhances decidualization of the remaining stromal cells [5], and quercetin—which possesses that senolytic activity—enhances decidualization through AKT–ERK–p53 signaling at 25 µM [4]. Whether such an intervention helps or harms therefore turns on the senescent burden at the moment of exposure, which is phase-dependent.

Table 3 compares these studies and the other principal primary sources on which this review’s biological claims rest. Two features of the table are deliberate. The first is the final column, which records the level of the outcome measured—molecular marker, cell adhesion, in vitro development, in vivo fertility, or clinical endpoint—because the distinction is routinely lost when such studies are summarized together. Of eleven entries, one reports a human pregnancy outcome [86], one reports fertility in mice [89], and one reports a clinical endpoint that is not a fertility endpoint [90]; the remaining eight stop at a molecular marker, an adhesion index or a cell-function measure. Claims in this field, including most of those advanced here, therefore rest on marker-level and adhesion-level evidence, and should be read accordingly. The second is that no entry reports a measured concentration in endometrial tissue, which is the review’s central observation shown at the level of the individual study rather than asserted in aggregate.

**Table 3:** Critical comparison of the principal primary studies underpinning this review. “Outcome level” classifies the highest-level endpoint actually measured. No study in this table reports a concentration measured in endometrial tissue. Cells were populated from the full text where a legally accessible open-access version existed and from the published abstract otherwise; “not stated in the published report” marks a value that neither source made available, and is distinguished from a quantity the study did not measure, which is marked as such. Values taken from the extraction table of a systematic review rather than from the primary report are identified in the cell concerned. Bold in the “Outcome level” column marks the three entries that reach beyond a molecular marker or adhesion index to a fertility, pregnancy or clinical endpoint.

Study	Model and System	n or Replication	Compound and Formulation	Concentration or Dose	Duration	Comparator	Main Result	Outcome Level	Key Limitation
[2]	Primary human endometrial stromal cells	8 independent primary cultures	Resveratrol, free	100 µM	4 or 8 days, concurrent with differentiation	Vehicle, decidualized	Represses *PRL* and *IGFBP1*; abrogates decidual senescence; accelerates *CRABP2*–RAR downregulation	Molecular marker	Single high concentration; in vitro; no dose–response
[3]	Primary and immortalized human endometrial stromal cells; biopsies from healthy cycling women	Not stated in the published report	Resveratrol, free	6.25, 12.5, 25, 50 µM (concentration series and timing from a secondary source [85])	Added at days 3 and 5 of decidualization	Decidualization cocktail alone	Dose-dependent increase in *PRL* and *IGFBP1*; reduced proliferation	Molecular marker	Highest concentration tested is half the single concentration used in the row above; cell source and stimulus also differ
[86]	Women undergoing IVF–embryo transfer; single-center retrospective cross-sectional cohort	204 cycles in 102 women (resveratrol) vs. 7073 cycles in 2958 women (control), from 8686 cycles screened	Resveratrol, oral supplement	200 mg/day, continuous	Throughout the transfer cycle, spanning the implantation window	Non-supplemented cycles, matched on age, embryo grade and stage, number transferred, and fresh vs. vitrified-warmed	Clinical pregnancy odds ratio 0.539 (95% CI 0.341–0.853); miscarriage odds ratio 2.602 (95% CI 1.070–6.325)	**Clinical pregnancy and miscarriage**	Retrospective and observational, so residual confounding by indication is not excluded; single center; supplement composition and adherence not verified; no exposure measurement
[21]	Ishikawa cells; human endometrial xenograft in RAG-2 mice	Not stated in the published report	Resveratrol, free; subcutaneous pellets in vivo	Low vs. high in vitro; 6, 30, 60 mg pellets	30 days in vivo	Estradiol; estradiol + progesterone	Estrogen agonist at low and antagonist at high concentration; reduced *ESR1* and Ki-67 at 60 mg	Molecular marker; tissue proliferation	Cancer cell line and xenograft; proliferative rather than decidual endpoints; no tissue assay of resveratrol
[4]	Menstrual effluent-derived endometrial stromal cells from unaffected controls and endometriosis patients, passages 1–3	*N* = 3–5 per condition (decidualization assays); *N* = 4–6 (viability)	Quercetin, free	25 µM for the decidualization assays; 0–50 µM dose–response	4 h pretreatment before cAMP (0.5 mM) ± medroxyprogesterone acetate (10^−7^ M); 72 h for the dose–response	Vehicle, basal and decidualized	Promotes decidualization (*IGFBP1*, *PRL*) via AKT–ERK–p53; proliferation reduced from 25 µM	Molecular marker	In vitro; replication at the level of conditions rather than donors; patient-derived cells of mixed origin
[5]	Human endometrial stromal cells	Not stated in the published report	Quercetin and dasatinib (senolytic)	Not stated in the published report	Not stated in the published report	Vehicle	Reduces senescence markers; increases *IGFBP1*, *PRL*, *FOXO1*	Molecular marker	In vitro; combination agent; concentration not stated in the published report
[72]	Human endometrial cells, in vitro implantation assay	Not stated in the published report	EGCG in menstrual-blood-derived exosomes (86.2 nm; −11.8 mV; 65.18% encapsulation)	Not stated in the published report	Not stated in the published report	Free EGCG	Greater adhesion; stronger upregulation of *LIF*, *HOXA10*, integrin β3 than free compound	Cell adhesion	In vitro; no endometrial concentration; no embryo assessed
[17]	Human endometrial cells, in vitro implantation assay	Not stated in the published report	Sildenafil citrate in RGD-functionalized nanostructured lipid carrier (92.7 nm; PDI 0.21)	Not stated in the published report	Not stated in the published report	Free drug; unfunctionalized carrier	Attachment increased 1.6 ± 0.4, 1.7 ± 0.3 and 2.3 ± 0.3-fold; *LIF* and *VEGF* upregulated	Cell adhesion	Not a phytochemical; in vitro; no concentration; no embryo assessed
[91]	Washed human spermatozoa suspended in Ham’s F10, in vitro	Not stated in the published report	Curcumin, free	30 µg/mL and 300 µg/mL	30–60 min	Vehicle	30 µg/mL: 20% loss of motility within 30 min without loss of viability. 300 µg/mL: >50% loss immediately, total loss within 60 min. Mitochondrial transmembrane potential reduced at all doses	Cell function	In vitro; prefertilization endpoint; sample size not reported
[89]	Human and murine spermatozoa in vitro; mouse fertility in vivo	Not stated in the published report	Curcumin, including intravaginal administration	Concentration-dependent; complete block at the higher concentrations	Complete block of motility within 5–15 min in vitro	Vehicle	Concentration-dependent loss of forward motility, capacitation/acrosome reaction and murine IVF; significant reduction in fertility in vivo (*p* < 0.001), most marked intravaginally; reversible	**In vivo fertility**	Murine; prefertilization mechanism; not an implantation endpoint; concentrations not stated in the published abstract
[90]	Women with symptomatic fibroids; double-blind placebo-controlled randomized pilot trial	39 recruited, 33 completed (22 treated, 11 placebo)	Green tea extract (45% EGCG), oral	800 mg/day	4 months	Placebo (800 mg brown rice)	Fibroid volume −32.6% vs. +24.3% with placebo (*p* = 0.0001); symptom severity −32.4% (*p* = 0.0001)	**Clinical endpoint**	Pilot size; fibroid rather than fertility endpoint; oral route with no tissue measurement

### 4.3. Consequence for the Physical Form: The Device Question

The route that maximizes local concentration is the route that most risks physical interference, and the strength of that claim must be stated carefully, because the obvious precedent does not support it. Intrauterine contraception was long assumed to act by preventing implantation, but current evidence indicates that intrauterine devices exert their primary effect before fertilization by reducing the opportunity for sperm to fertilize an ovum, and no scientific evidence establishes that prevention of implantation results from their use [49]. A separate review of emergency contraception reaches a comparable conclusion for hormonal methods with theoretical endometrial effects [92]; the two are distinct publications and are cited here as such.

The correct reading is therefore not that an intrauterine foreign body is known to block implantation, but that the question has not been settled and the risk has not been excluded. The distinction between physical forms is part of what has not been settled, and collapsing those forms overstates the case. A transient intracavitary infusion, which occupies the cavity for minutes, a retained hydrogel, which occupies it for days, and an indwelling device, which occupies it indefinitely, present different mechanical propositions, and nothing in the retrieved literature compares them for implantation. The timing of administration separates equally cleanly: a formulation instilled before embryo transfer and cleared before it takes place is a different proposition from one present in the cavity while an embryo is attaching, and only the second raises the question at issue here.

What supports treating the risk as a real design constraint is the pattern of practice rather than a demonstrated mechanism: every intrauterine system reviewed below is deployed outside the implantation window. Intrauterine hydrogel and biomaterial systems have been developed for endometrial regeneration—an exosome-bearing chitosan/glycerophosphate system attenuating fibrosis in a murine intrauterine adhesion model [50], an antioxidant hyaluronan/chitosan hydrogel carrying platelet-rich plasma and stem cells to restore fertility [51], dual-network hydrogels for locationally activated platelet-rich plasma [93], glycyrrhizic acid-based growth factor hydrogels [94], and engineered extracellular vesicles delivered for gene therapy of intrauterine injury [95]. In every case the carrier is placed into a damaged uterus to induce regeneration, not into a receptive cavity coexisting with an embryo—which is precisely the configuration in which the risk would have to be evaluated, and has not been. The clinically adjacent practice—intrauterine infusion of growth factors or platelet-rich plasma for thin endometrium—is performed before transfer rather than around it, and its efficacy remains contested [96,97].

The proposition can then be stated at the strength the evidence supports. As a hypothesis requiring evaluation, the route that maximizes endometrial exposure may be the route that most interferes with implantation, so that exposure and implantation are opposed along the same axis. Both of its components are unverified. The ranking of routes by local concentration has not been measured for any phytochemical; the interference term has not been measured at all in a receptive cavity. It is presented here as a design risk to be tested—the experiment is a comparison of implantation rates with and without an intracavitary formulation of defined residence time, in a model that permits implantation—and not as an established trade-off. Table 1 and Figure 1 mark it accordingly.

### 4.4. Consequence for the Compound: Its Own Duality

Even a safe carrier does not remove the problem, because increasing delivery can itself enter a harmful regime. Curcumin is the clearest case: it immobilizes human sperm in a concentration- and time-dependent manner [91], and in a murine model concentration-dependent loss of sperm motility, capacitation and in vitro fertilization was accompanied by a significant reduction in fertility in vivo, most pronounced when the compound was administered intravaginally [89]. The antifertility effect was reversible [89]. The mechanism is prefertilization rather than anti-implantation, but the implication for delivery design is direct: the compound and the route that this review proposes for receptivity support are the same compound and route that have been advanced as a contraceptive.

The pattern is not confined to curcumin. In endometriotic and eutopic endometrial cells, curcumin suppresses angiogenic and proliferative signaling and reduces estradiol production [98,99]—activities that are therapeutic in endometriosis and are not obviously desirable in an endometrium preparing for implantation. Epigallocatechin gallate makes the same point from the opposite direction. In a pilot placebo-controlled randomized trial, four months of oral green tea extract reduced fibroid burden and improved symptom scores in women with symptomatic uterine fibroids [90]; a multicenter randomized trial of the compound in women with fibroids and unexplained infertility is now underway, and its published protocol frames the compound as a fertility intervention [100], with hepatic safety in reproductive-aged women assessed separately [101]. The same compound is therefore being advanced simultaneously as an antiproliferative agent and as a fertility adjunct. The completed pilot trial also qualifies the argument of Section 2: an oral route with unfavorable systemic pharmacokinetics has nonetheless produced a clinical signal in uterine tissue, which shows that oral administration is not inert and that the objection raised here is interpretive rather than absolute—without an endometrial concentration the observed effect cannot be assigned to a concentration at the tissue.

The generality of biphasic behavior should be stated more carefully than it often is. Hormesis is a well-documented general property of plant-derived bioactives [102], and for resveratrol a concentration-dependent reversal of direction has been shown in an endometrial cell line, accompanied by a single-direction effect at the highest dose in a human endometrial xenograft [21]. It does not follow that every compound discussed here has a biphasic dose–response, and no such curve has been constructed for any of them at the endometrium. In particular, opposing results obtained in different studies are not by themselves evidence of a biphasic curve, since the studies differ in more than concentration (Table 3). What the available evidence does support is narrower and sufficient for the design argument: for at least one of these compounds, direction of effect changes with concentration in an endometrial cell system [21], the two in vitro studies that disagree about its effect on decidualization differ two-fold in concentration (Section 4.2), the one human cohort that examined continuous oral exposure across the window reports an association with reduced clinical pregnancy and increased miscarriage [86], and for none of them is the endometrial concentration known. Under that combination, raising exposure is not automatically beneficial, and the design objective is bounded exposure within an interval that has been measured rather than maximal exposure.

### 4.5. Synthesis: A Context-Dependent Set of Design Choices

The requirements that follow are design choices contingent on setting and on evidence status, not a general reversal of drug-delivery principles, and Table 4 is written to make both dependencies explicit. Three of the five entries are ordinary targeting objectives applied to an unusual target; two are specific to the presence of an embryo and have no counterpart in conventional practice. The right-hand columns state the setting in which each applies and the evidence on which it rests, so that a reader can see which entries are established and which are proposed.

**Figure 2:**
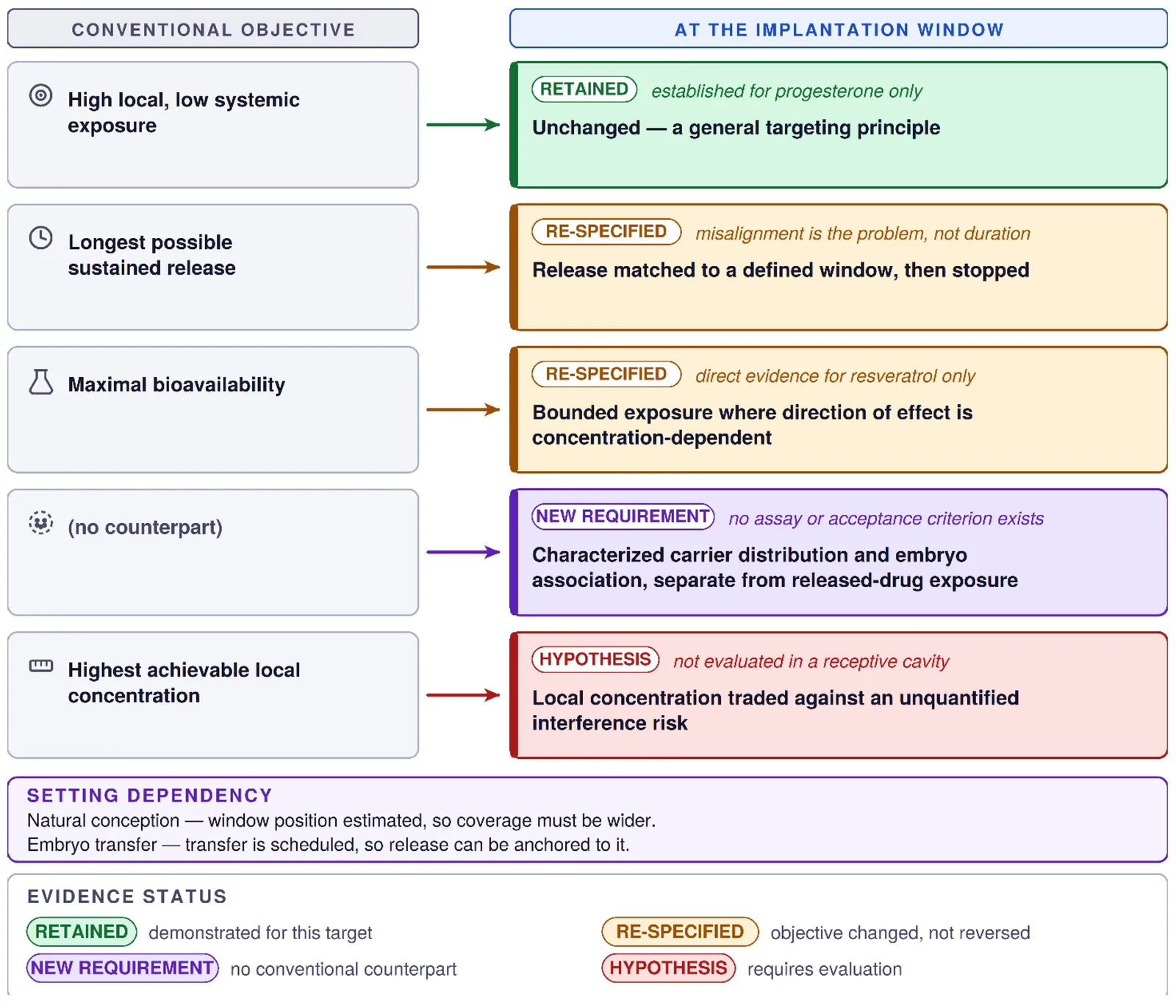
Conventional delivery objectives and their peri-implantation counterparts. Maximal bioavailability, longest sustained release and highest local concentration—conventional figures of merit—are re-specified rather than reversed at this target: release is matched to a defined window rather than extended indefinitely, exposure is bounded rather than maximized where direction of effect is concentration-dependent, and local concentration is traded against a physical-interference risk that has not been quantified. Characterized carrier distribution and embryo association—assessed separately from the exposure of the embryo to the released compound—is a requirement with no counterpart in conventional practice and no current standard. Each entry carries its evidence status: RETAINED where the objective is unchanged, RE-SPECIFIED where it is redefined rather than reversed, NEW REQUIREMENT where conventional practice has no counterpart, and HYPOTHESIS where evaluation is still required.

Figure 2 presents the same choices as a comparison with conventional objectives. Peri-implantation delivery therefore requires a partial and conditional re-specification of the objective function of conventional delivery engineering—maximal bioavailability, longest duration, highest local concentration—rather than its wholesale reversal. The reviews identified in Section 1.1 do not formalize these dependencies; doing so is the contribution of this section.

## 5. Quantifying Exposure: What Must Be Measured

**Figure 3:**
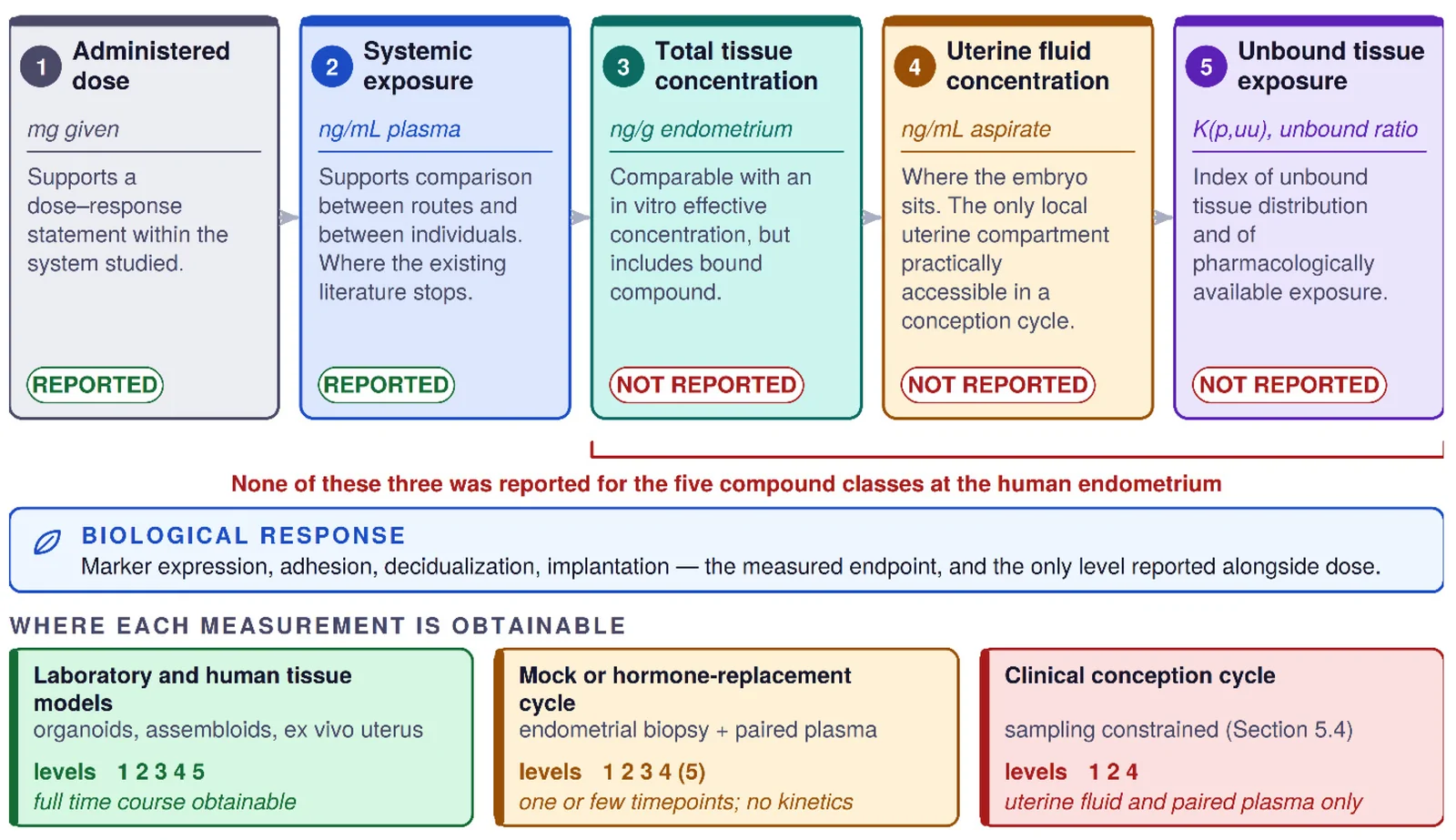
The exposure cascade from administered dose to biological response, and what each level supports. An administered dose supports a dose–response statement within the system studied. Systemic exposure supports comparison between routes and between individuals, and is the level at which the existing phytochemical pharmacokinetic literature stops (Section 2). Total tissue concentration supports comparison with an in vitro effective concentration, but includes bound compound and so overstates the pharmacologically active fraction. Uterine fluid is the compartment in which an embryo is suspended, and the only local uterine compartment that is practically accessible in a conception cycle. Unbound tissue exposure—the ratio of unbound tissue to unbound plasma concentration—is an index of the extent of unbound tissue distribution and of the pharmacologically available exposure at the target. For the five compound classes at the human endometrium, none of the three levels above systemic exposure was reported in the retrieved literature. The lower band shows where each measurement is obtainable: laboratory and tissue models, mock or hormone-replacement cycles, and clinical conception cycles, in which only uterine fluid and paired plasma can be sampled without harm.

Section 1 distinguished five quantities that are commonly collapsed into “dose”: the administered dose, systemic exposure, total tissue concentration, uterine fluid concentration, and the pharmacologically available (unbound) exposure at the target. Figure 3 sets out that cascade, together with what each level supports and the study settings in which it is obtainable. The rest of this section applies the cascade to the endometrium.

### 5.1. Endometrial Tissue Concentration Is Measurable—And Has Been Measured for One Compound

The demonstrations assembled in Section 3.3 settle the methodological question. Endometrial tissue concentrations have been quantified in human tissue for vaginally administered progesterone in two studies—an ex vivo perfusion model [23] and a randomized comparison in postmenopausal women [24]—summarized against systemic administration in a third [25], and shown to be both disproportionate to serum levels and dependent on cycle phase. The frequently co-cited vaginal-tablet pharmacokinetic study reports plasma concentrations only [26], and the general review of the intravaginal route [54] summarizes the phenomenon rather than adding a measurement; neither is a source of tissue concentration data, and neither is cited as such here. Stimulus-responsive release platforms capable of confining exposure to a defined interval are likewise available in principle [103].

### 5.2. What Is Not Measured

For every phytochemical proposed for endometrial support, none of the following was reported for women in the literature retrieved: endometrial tissue concentration after any route; the ratio of endometrial to plasma concentration; the unbound fraction in endometrial tissue or uterine fluid; the time course of endometrial exposure relative to the implantation window; the chemical species present in the tissue as opposed to the plasma; and the dependence of any of these on cycle phase. The screening described in Section 1.2 quantifies this: of 105 unique records combining one of the five compound classes with endometrial and concentration terms, exactly one reported a measured tissue concentration of a study compound—total genistein in whole rat uterus after lifetime dietary exposure [18]—and none reported an endometrial tissue or uterine fluid concentration in women. The near-misses are instructive about where the gap lies. One randomized trial sampled endometrial tissue, endometrial fluid and blood in women taking oral resveratrol, and measured MMP-2 and MMP-9 rather than the compound [19]. The others measured endometrial gene and protein expression [20], receptor and proliferation endpoints after dosing [21], or urinary rather than tissue concentrations [22]. Sampling access is therefore not the obstacle; the analyte chosen is. That set of absences is not a minor gap in a mature field. It is an unmeasured variable across the studies retrieved in this class.

### 5.3. The Contrast with the Timing Axis

The comparison is instructive because the adjacent literature has developed a quantitative apparatus for a different variable. Endometrial receptivity is now assessed by transcriptomic profiling, with displacement of the window of implantation described as a distinct clinical entity [104,105,106] and demonstrable under defined physiological conditions [107]; conventional and modern markers of receptivity have been compared systematically [75]. The availability of such a test and its demonstrated clinical usefulness are different questions, and the second remains unsettled: personalized transfer timing based on these assays has not shown consistent benefit in randomized or pooled analyses [108,109], and the failure of euploid blastocysts to implant has multiple candidate explanations of which endometrial asynchrony is only one [110]. In parallel, recurrent implantation failure has been subjected to consensus definition and good-practice recommendation [111,112,113].

The asymmetry is therefore in what has been built rather than in what has been proven. For the timing axis the field has constructed standardized, commercially available and actively contested assays, and is now arguing about whether acting on them improves outcomes—a debate that presupposes a measurement. For the exposure axis there is no equivalent measurement to argue about. A proposal for the latter is set out in Section 7.1.

### 5.4. The Sampling Limit and the Case for Human Tissue Models

Serial endometrial sampling through the implantation window of a conception cycle is not ethically available, which places a hard ceiling on the in vivo characterization of exposure kinetics in exactly the cycle that matters. Any complete time course must therefore be reconstructed from one of three substitutes, and their trade-offs are worth stating.

The first is the non-conception cycle. Single-timepoint biopsies in mock or hormone-replacement cycles are obtainable, and were the basis of the design used in the randomized vaginal-gel comparison of Section 3.3 [24]; they give a defensible concentration but not a kinetic profile, and they cannot report what an embryo present in the cavity would have changed. The second is the ex vivo perfused human uterus, which yielded the diffusion time course and the cycle-phase dependence cited above [23]; it preserves human tissue architecture and permits serial measurement, but only for as long as the preparation remains viable. The third is the animal model, which allows true serial sampling at the cost of the extrapolation limit set out in Section 3.5.

A fourth substitute is available in a conception cycle and is the only one that is: aspiration of endometrial fluid immediately before embryo transfer is performed routinely and non-invasively in assisted reproduction, and has been used to characterize the receptive and non-receptive endometrium proteomically in a cohort of 110 women [83]. That this is compatible with phytochemical dosing is not hypothetical: paired endometrial tissue, endometrial fluid and blood have already been collected before and after a 12–14-week course of oral resveratrol in a randomized trial, with the compound left unmeasured [19]. Uterine fluid is not endometrial tissue, and the relationship between the two compartments has not been established for any phytochemical; but it is a samplable compartment in the cycle of interest, and it is the compartment in which an embryo is actually bathed. Section 7.1 therefore treats it as a distinct measurand rather than as a proxy for tissue.

None of these delivers, on its own, a human peri-implantation time course with an embryo present. That combination is what human tissue models are for, and it is the principal task assigned to them in Section 7.3.

## 6. Class-Specific Delivery Strategies

Section 2 established that the five compound classes are limited at different points along the route from ingestion to tissue. It follows that the delivery intervention differs by class, and that a formulation succeeding for one class carries no presumption of transfer to another. Table 5 restates the dominant failure points as design targets.

Two columns have been added to the table relative to the framework of Section 2, and they carry the qualifications that the framework itself cannot. The “also present” column records the other processes that operate in each class besides the dominant barrier, so that the table is not read as assigning one obstacle exclusively to each class; where a listed process would increase rather than reduce exposure, it is identified as such. The “basis and generalizability” column records the species and the compound in which each assignment was established, so that a reader can see where the evidence is human and where it is not, and which assignments rest on a single compound rather than on the class.

Two features of this table deserve emphasis. First, each entry is a distinct testable hypothesis rather than a general aspiration, and each specifies the measurement that would refute it—in every case, an endometrial concentration of the parent compound, or of the intended species, obtained after the proposed intervention and compared with that obtained after the oral route. Second, the strategies are not equally novel: the flavonoid entry has a direct in vitro precedent [72], the carotenoid entry has a formulation precedent outside reproduction [32], and the phytoestrogen entry requires no new delivery technology at all, only substitution of the administered species.

A third feature should be stated because it limits all five. None of these interventions has been tested at the endometrium, and the table is therefore a research agenda rather than a set of recommendations. The order in which the entries could be attempted follows from the preceding column: the phytoestrogen entry is immediately testable with existing materials, the flavonoid entry has a carrier already demonstrated in vitro, and the curcuminoid and stilbene entries require formulation development before an exposure measurement would be meaningful.

## 7. What Would Have to Be Built

### 7.1. A Minimum Reporting Standard for Endometrial Exposure

The standard proposed here distinguishes three measurands that are frequently reported as though they were one, and scales what is asked of a study to what its design permits.

#### 7.1.1. The Three Measurands

(i) *Tissue homogenate concentration*—the total amount of compound per unit mass of endometrial tissue, reported in ng/g or nmol/g of wet tissue with the wet-to-dry ratio stated. It is the measurand of the progesterone data in Table 2 and is the most directly comparable with an administered dose, but it includes protein- and lipid-bound compound and therefore overstates the pharmacologically active fraction. (ii) *Uterine fluid concentration*—the amount per unit volume of aspirated endometrial fluid, reported in ng/mL with the aspirated volume, any dilution, and analyte recovery from the aspiration device stated. The fluid is the compartment in which an embryo is suspended, and the only local uterine compartment that is practically accessible in a conception cycle [83]. (iii) *Pharmacologically available exposure*—the unbound concentration at the target, expressed either as the unbound fraction in endometrial tissue or, preferably, as the ratio of unbound tissue to unbound plasma concentration (K_p,uu_), which is the best available index of the extent of unbound tissue distribution and therefore of the pharmacologically available exposure at the target [6]. It can be approached by equilibrium dialysis of homogenate, by tissue slices, or in vivo by microdialysis, which samples the unbound interstitial compartment directly and is the only clinical technique that distinguishes bound from unbound and parent from metabolite [7].

#### 7.1.2. The Reporting Items

A study proposing a phytochemical for endometrial receptivity should report the route, formulation and administered dose; the measurand determined, in explicit units; the time of sampling relative both to the dose and to the implantation window; a plasma concentration obtained at the same timepoint, so that a tissue-to-plasma ratio can be formed; the chemical species quantified, distinguishing parent compound from conjugates and microbial metabolites; the analytical method with its lower limit of quantification and validated range; analyte recovery and stability in the relevant matrix, together with any validation of the homogenization or extraction procedure; and the hormonal state of the endometrium at sampling, by cycle phase or by the exogenous regimen used. Where the vaginal route is used, the local microbial variable identified in Section 3.5 should be reported alongside the rest.

#### 7.1.3. Proportionality to Design

The standard is a minimum dataset for interpretability, not a methodological prescription, and what is feasible differs by setting. In laboratory and human tissue models, all three measurands are obtainable, together with a time course, and nothing less should be accepted. In mock or hormone-replacement cycles, tissue homogenate concentration, uterine fluid concentration and paired plasma are obtainable at one or a small number of timepoints, but a kinetic profile is not; the single-timepoint limitation should be stated rather than smoothed over. In a clinical conception cycle, the sampling constraint of Section 5.4 is binding: uterine fluid aspirated immediately before transfer, with paired plasma, is the realistic maximum, and endometrial tissue is not obtainable. Outside a conception cycle the constraint is weaker than is often assumed—paired endometrial tissue, endometrial fluid and blood have already been collected before and after oral dosing with a phytochemical in a randomized trial [19]—so for a non-conception study the limiting factor is the decision to assay the compound, not access to the compartment. A study should report what its design permitted and state explicitly which measurands it could not obtain, rather than reporting an administered dose alone.

The standard is not aspirational in the sense of requiring new methods. Every element has been demonstrated—tissue concentration for progesterone in human endometrium (Section 3.3), uterine fluid sampling in a clinical IVF cohort [83], and unbound tissue measurement across other organs [6,7]—and every element is measurable with existing analytical chemistry. What has not been done is their application to this compound class at this tissue.

### 7.2. A Dual-Readout Screen for Carrier Safety

The gap identified in Section 4.1 is specific enough to be closed by a defined assay. What is required is a screen with two simultaneous readouts: endometrial delivery efficiency on the one hand, and characterized exposure of the preimplantation embryo on the other. The second readout is the novel element, and the existing evidence indicates both that it is necessary and how it could be built—lipid nanoparticle penetration of the zona pellucida varies with stage of exposure and with surface composition [81], so surface charge and lipid composition are the natural screening axes.

What follows is a specification for an assay that does not yet exist. It is offered as a starting point for methodological discussion, not as a validated standard, and no system has been characterized against it.

#### 7.2.1. Four Readouts, Not One

Following Section 4.1, the screen must report carrier distribution, released-drug exposure of the embryo, effect on the endometrial compartment, and developmental outcome as separate results. A system that excludes the carrier but delivers the payload to the embryo has failed, and a single composite verdict would conceal that.

#### 7.2.2. Acceptance Criteria

Five requirements are proposed, with the statistical reasoning made explicit because the alternatives are easy to state and unsound.

#### 7.2.3. Carrier Distribution

Carrier-associated signal within the embryo below a stated, validated limit of detection, with the limit reported in interpretable units—particles per embryo, or mass of label per embryo—and with the method’s single-particle sensitivity established in the same matrix. A result below the limit of detection bounds carrier entry at that limit; it does not demonstrate absence, and should be reported as “below X particles per embryo” rather than as “no entry”. Positive controls must include a nanoparticle-sized labeled species, because zona permeability to a labeled macromolecule [82] establishes that the barrier is penetrable but does not validate detection of an intact nanocarrier; the two differ in size, in label density and in optical behavior. Negative and specificity controls must include free label alone, empty carrier alone, and the released drug alone, so that a signal can be assigned to the intact particle rather than to dissociated label. A program should also state in advance the level of carrier entry it will treat as disqualifying, and justify that level against the concentration at which the payload affects development in the same system, rather than reporting the detection limit alone.

#### 7.2.4. Released-Drug Exposure

The payload concentration reaching the embryo or its immediate medium, measured directly rather than inferred from carrier exclusion, and compared with the concentration at which the compound affects development in the same system.

#### 7.2.5. Endometrial Effect

A predefined set of endometrial readouts—decidualization markers, uterine fluid composition, and the embryo-selection response of (iii) in Section 4.1—compared with vehicle against margins set in advance and reported separately from the developmental outcome, since a formulation can leave the embryo untouched and still alter the environment in which it implants.

#### 7.2.6. Developmental Outcome

Equivalence to vehicle within a predefined margin rather than absence of a statistically significant difference. The margin should be set in advance—for example, a difference in blastocyst formation rate of no more than a stated number of percentage points, and a difference in total or inner-cell-mass cell number of no more than a stated proportion—and the two-sided confidence interval for the observed difference must lie entirely within it. The sample size must be calculated to give adequate power against that margin, and must be reported. A non-significant difference in an underpowered experiment is not evidence of equivalence, and should not be presented as a safety result.

#### 7.2.7. Exposure Conditions

All four readouts obtained at the highest carrier concentration the intended endometrial dose would produce in the cavity, for a duration spanning the intended delivery interval, rather than at a nominal fraction of either. Each is obtained separately at the three exposure configurations the embryo passes through—zona-intact, post-hatching trophectoderm, and post-attachment—because the barrier differs at each (Section 7.3).

None of the systems reviewed in Section 3.6 has been characterized against criteria of this kind, and the criteria themselves would require validation—in particular, agreement between the proposed readouts and outcomes in a reference set of carriers of known behavior—before they could be treated as a standard.

### 7.3. Evaluation in Human Tissue Models

The measurements called for above cannot all be made in vivo (Section 5.4), and the relevant human tissue models have matured rapidly. Endometrial organoids recapitulate epithelial physiology and hormone responsiveness [114,115], and can be maintained in fully synthetic matrices that remove a major source of experimental variability [116]. Assembloids combining epithelial and stromal compartments model decidual transformation and its senescence dynamics [88,117], and have been extended to recapitulate the embryo–endometrial interface [118]. On the embryonic side, blastoids model blastocyst development and implantation without requiring human embryos [119], and integrated in vitro systems for human implantation and implantation failure have now been reported [120]. Trophoblast organoids provide the corresponding placental compartment [121].

Three qualifications govern how these platforms can be used for the safety question, and they are stated here because the attraction of the models makes it easy to overstate what they deliver.

#### 7.3.1. The Barrier Changes with Developmental Stage, and the Zona Is Only the First

Before hatching, a carrier must cross the zona pellucida to reach the embryo, and that is the barrier the lipid nanoparticle data address [81,82]. At hatching, the zona is shed and the trophectoderm surface is directly exposed to the cavity, which removes the barrier rather than replacing it. After attachment, the relevant interface is invading trophoblast in contact with maternal tissue, and the question becomes one of uptake and developmental effect rather than of penetration. A screen framed around zona penetration therefore addresses only the first of three configurations, and a carrier present in the cavity across the window will encounter all three. Each stage requires its own exposure and its own readout.

#### 7.3.2. Each Model Reproduces Part of This and Should Be Used for That Part

Endometrial organoids and assembloids reproduce the maternal compartment—epithelial barrier function, hormone responsiveness, decidual transformation—and are appropriate for delivery efficiency, for tissue concentration and for the endometrial-effect readout of (iii) in Section 4.1; they contain no embryonic structure and cannot report penetrance. Blastoids reproduce aspects of blastocyst architecture and attachment behavior and can report carrier association with an embryo-like structure; whether they reproduce the zona pellucida and its permeability depends on the specific protocol and must be established rather than assumed, and a blastoid without a zona cannot be used to test a zona-dependent barrier. Embryo–endometrial interface models [118] and integrated implantation systems [120] reproduce the apposition step and are the appropriate setting for the attachment-stage configuration.

#### 7.3.3. Blastoids Are Not Validated Equivalents of Embryos for Safety Assessment

They are research models of specific phenotypic features of the embryo, and their concordance with human embryos has not been established for particle uptake, for barrier permeability or for developmental toxicity. Before a blastoid readout could substitute for an embryo readout in a safety decision, concordance would have to be demonstrated across a reference set of carriers spanning known penetrant and non-penetrant behavior, and the limits of that concordance stated. Until then, blastoids are appropriate for screening and for mechanism—ranking carriers, identifying surface determinants, generating hypotheses—and not for establishing that a carrier is safe. The honest summary is that these models can measure carrier association, payload distribution and gross developmental endpoints in a human-derived system; what remains uncertain is how those measurements map onto a human embryo in vivo.

Work with stem cell-based embryo models is subject to its own oversight, which is model-specific rather than inherited from embryo research, and which has been revisited as the models have advanced [122]. Any program of the kind proposed here would be designed within that framework and under the applicable national regulation, and the oversight requirements differ among endometrial organoids, assembloids and stem cell-based embryo models; they should be addressed separately for each.

These platforms make the two required measurements tractable together: exposure kinetics in endometrial tissue, and carrier association with an embryo-equivalent structure. Assembling them into a delivery-evaluation platform is a distinct undertaking; what is specified here is the set of measurements such a platform would have to make, and the validation it would itself require before its results could be extrapolated to human exposure or developmental safety.

### 7.4. Translational and Regulatory Considerations

Two features of this compound class complicate translation, and the regulatory statement should be made jurisdiction by jurisdiction rather than in general.

First, the products are consumed under a regulatory framework that differs from that applied to medicines, and the specifics differ by country. In the United States, dietary supplements are marketed under a framework separate from drug approval, and the scientific and regulatory challenges this creates for establishing safety, quality and efficacy have been described in detail, including the difficulty of characterizing the actual composition of marketed products. The same source notes that these challenges are not confined to one jurisdiction, as the market has become global [123]. In the Republic of Korea, where the authors are based, equivalent products are marketed as health functional foods under a statutory framework separate from that governing pharmaceuticals. The relevant point for this review does not depend on the details of any one scheme: a product consumed under a food-supplement framework has not been characterized to the standard that would allow its endometrial exposure to be inferred, and its use is not captured in the clinical record. Use of complementary therapies and of dietary supplements is common in exactly the populations undergoing fertility care [8,9], so the counterfactual for any trial is not an unexposed population but an uncharacterized one. A related and separate hazard is pharmacokinetic interaction with conventional drugs, which has been reviewed specifically for the curcuminoids [124] and which applies in a setting where patients are concurrently receiving gonadotropins and steroids.

Second, a vaginal or intrauterine formulation of a compound currently sold as a food supplement is a drug product in every jurisdiction considered here, and its development carries the regulatory burden of one—chemistry and manufacturing controls, and reproductive and developmental toxicology under the applicable guideline, which for an agent intended for use around conception would include the fertility and early embryonic development study described in ICH S5(R3). The embryo-safety package described in Section 7.2 is not a current regulatory requirement, and should not be represented as one. It is an additional measurement the authors argue is scientifically necessary for this particular target, and the distinction between what regulators require and what this review proposes is maintained deliberately.

Neither point argues against the program proposed here. Both argue that its first products should be measurements rather than interventions: the endometrial exposure of compounds already being taken, and the embryo-penetrance profile of carriers already in use. Those are tractable now, and they are prerequisites for any responsible trial.

## 8. Conclusions

Whether a given phytochemical helps or harms endometrial receptivity is settled by context, and one axis of that context—concentration at the tissue—is missing from the literature this review retrieved, with a single rat whole-uterus measurement as the nearest exception [18]. The consequence should be stated at the strength the evidence supports. The omission does not invalidate individual studies, which test an administered dose against an outcome within the system they use. What it prevents is placing results from different systems on a common concentration scale, connecting an in vitro effective concentration to an achievable in vivo exposure, and attributing a directional reversal to concentration rather than to the design differences that usually accompany it. Together with differences in timing, tissue state and endpoint, the missing measurement is a principal reason the efficacy literature has not converged.

Three conclusions follow. First, “poor oral bioavailability” is several overlapping problems rather than one, and because the rate-limiting step differs by compound class, no single formulation strategy addresses them; each class instead defines a specific and testable intervention. Second, endometrial targeting is neither speculative nor unprecedented—the first uterine pass is quantified in human tissue for progesterone, vaginal formulation platforms are established for other indications but tuned to release over weeks rather than days (Section 3.4), and uterine and placental nanocarriers exist at a preclinical stage, with receptivity-directed carriers demonstrated in vitro [17,72]—but none of this work has been carried out during an implantation window, with a measured exposure, or with any assessment of the embryo. Third, that target imposes a constraint not typically encountered in conventional tissue-targeted delivery. The tissue contains an embryo that lipid nanoparticles can reach, so carrier distribution must be verified, and, separately, so must the exposure of the embryo to the released compound, the effect on the endometrial environment, and developmental outcome. And a delivery system precise enough to support implantation is, by the same token, precise enough to oppose it: intravaginal curcumin has been advanced as a contraceptive [89], and the single human cohort examining continuous oral resveratrol across embryo-transfer cycles reports an association with reduced clinical pregnancy and increased miscarriage rather than with benefit [86]. What follows is that bounded exposure, rather than maximal exposure, is the design objective wherever direction of effect is concentration-dependent.

This review has limitations of its own. It is a narrative review built on a structured search rather than a systematic review with quantitative synthesis, and carries the selection risk that this entails; the search is documented in full in Section 1.2 and the Supplementary Material so that this risk can be assessed rather than assumed. The search was restricted to English-language records indexed in PubMed, which will underrepresent literature on traditional preparations published elsewhere, and in particular the East Asian herbal medicine literature, in which several of these compounds have a long history of use. The five compound classes were chosen by the criterion given in Section 1.2, and other classes may prove equally relevant once their pharmacokinetics are characterized. The assignment of a dominant barrier to each class rests, for two of the five, on a single compound studied in a single species, and should be treated as provisional. Screening of the evidentiary search block was performed on complete abstracts rather than full texts (Section 1.2), so a tissue concentration reported only in a results table of a paper whose abstract does not mention it would not have been retrieved. Finally, the central negative claim concerns what a defined search retrieved, not a proof that no such measurement exists anywhere; it is also narrower than it is sometimes convenient to state, since one retrieved study does report a uterine tissue concentration of a compound in these classes, in the rat [18]. The control search described in Section 1.2 shows that the strategy retrieves endometrial tissue-concentration studies when they exist, which makes a query artifact an unlikely explanation; but a single unpublished or non-indexed dataset reporting endometrial concentrations would not overturn the argument, and it would sharpen it.

The practical consequence is a conditional re-specification of conventional objectives: release matched to a defined window in place of release maximized in duration, bounded exposure in place of maximal exposure where direction of effect is concentration-dependent, and separate verification of carrier distribution, released-drug exposure, endometrial effect and developmental outcome, for which no standard currently exists. The components required—quantified routes, carriers at proof-of-concept stage, human tissue models capable of the necessary measurements—are available. What is absent is their assembly toward this target, and the measurements that would make the resulting claims interpretable.

## Figures and Tables

**Table 4:** Design choices for peri-implantation delivery, with the setting in which each applies and its evidence status.

Design Choice	Basis	Relation to Conventional Delivery Design	Setting in Which it Applies	Evidence Status
High local, low systemic exposure	Minimize maternal exposure and exposure of non-target tissue	Consistent—a general targeting principle	All settings	Established for progesterone by the vaginal route (Table 2); not demonstrated for any phytochemical
Release profile matched to the window rather than maximized in duration	Section 4.2; exposure outside the intended interval has uncertain direction of effect	Context-dependent—sustained release engineered to cover the window is appropriate; exposure persisting beyond it is not	Natural conception: wider coverage, window position estimated. Embryo transfer: narrower coverage anchored to a scheduled event	Supported by a conflicting study pair whose difference is not attributable to timing alone (Table 3); hypothesis
Characterized carrier distribution and embryo association, assessed separately from released-drug exposure	Section 4.1	Novel requirement—no current standard	Any setting in which the formulation is present while an embryo is in the cavity	Entry into the embryo demonstrated for lipid nanoparticles [81]; no assay or acceptance criterion established
Minimal physical interference; residence time specified	Section 4.3	Novel requirement	Intrauterine route only; before-transfer administration largely avoids it	Hypothesis; not evaluated in a receptive cavity
Bounded rather than maximal exposure	Section 4.4	Context-dependent—applies where direction of effect is concentration-dependent	Compounds with demonstrated or suspected concentration-dependent reversal	Direct evidence for resveratrol [21]; general hormesis literature [102]; not established compound by compound

**Table 5:** Class-specific points of exposure failure and the corresponding points of delivery intervention. The dominant barrier is the step the available evidence identifies as rate-limiting; it is not the only barrier operating.

Class	Dominant Point of Exposure Failure	Also Present	Point of Delivery Intervention	Basis and Generalizability	Ref.
Curcuminoids	Quantity reaching the circulation (plasma curcumin below a 1 µg/mL limit of detection after 12 g orally—a bound on systemic exposure, not a direct measurement of absorption)	Intestinal and hepatic conjugation; chemical instability at intestinal pH; low aqueous solubility	Bypass absorption (local vaginal or intrauterine) or solubilized formulation; differentiate from the saturated nanoformulation literature by uterine targeting	Human, *n* = 10, single dose, curcumin; conjugation contribution inferred from a human piperine interaction study	[14,27,28]
Carotenoids	Parent compound not absorbed; only low crocetin appears	Metabolite-dependent activity; species difference not characterized	Bypass the gastrointestinal step at which the parent is lost, or deliver crocetin or a derivative directly (an intravenous crocetin derivative has human experience)	Rat, crocin only; should not be extended to other carotenoids or assumed to hold in humans	[30,32]
Flavonoids	Intestinal conjugation despite high permeability	Hepatic and renal elimination. Local deconjugation at the tissue would act in the opposite direction—a potential rescue rather than a barrier—and has been shown for resveratrol metabolites in endothelial cells, not for flavonoids at the endometrium	Bypass first pass (vaginal route) or conjugation-resistant formulation; the EGCG–exosome study is an in vitro precedent for a formulation-dependent endometrial response, not a demonstration of either	Murine single-pass perfusion for naringenin; human circulating-metabolite data for quercetin and kaempferol; qualitative finding transfers, proportions do not	[33,34,35,72]
Stilbenes	Combined intestinal and hepatic first-pass metabolism, with sulfate conjugation prominent; parent bioavailability <1% despite 75% absorption	Differential tissue partitioning of parent compound and metabolites; the intestinal component is shared with the flavonoids and is counted in the dominant barrier here rather than separately	Precise release kinetics aligned to the window plus metabolic stabilization; methylated analogs with improved bioavailability are a further possibility, not yet tested for this target	Human bioavailability data; rat tissue distribution; stabilized-analog rationale from the same review	[36,38]
Phytoestrogens	Host-dependent speciation of daidzein to equol (producers 30–40%)	Conjugation, as for the other polyphenols; no barrier specific to this class beyond speciation was identified in the retrieved literature	Deliver the active species (equol) directly, bypassing metabotype dependence	Human; specific to daidzein, not a property of genistein or of the class	[39,40]

## Data Availability

No new data were created or analyzed in this study. Data sharing is not applicable to this article.

## Supplementary Materials

The following supporting information can be downloaded at: https://www.mdpi.com/article/10.3390/ph19101620/s1, Table S1: Complete search strategy, retrieval counts and screening outcome; Table S2: Screening record for the 105 unique Block C articles.
