# Supplementary material for: Delivering Phytochemicals to the Implantation Window: The Dual Constraint of Achieving Endometrial Exposure Without Compromising the Embryo
Source: Pharmaceuticals (Basel). 2026 Oct 12;19(10):1620. doi: 10.3390/ph19101620 (PMC13708500; doi:10.3390/ph19101620)
Supplement: Supplementary file 1 [file pharmaceuticals-19-01620-s001.zip › pharmaceuticals-4591762-supplementary.pdf]

Supplementary Materials

Delivering Phytochemicals to the Implantation Window: The Dual Constraint of Achieving Endometrial Exposure Without Compromising the Embryo

Yohan Seo, Joohan Woo and Seong Woo Choi

Table S1. Complete search strategy, retrieval counts and screening outcome

All searches were run against PubMed through the NCBI E-utilities interface. Truncated terms are given unquoted, which is how they were executed, because PubMed does not guarantee wildcard expansion inside quotation marks. Every query carried the date limit `("1900"[PDAT] : "2026/05/31"[PDAT])`, shown as `[DATE]` below for readability and reproduced in full in the first row. Counts were obtained on **2 October 2026** and are exactly reproducible by re-executing the strings given. Blocks A and B were screened by title and abstract to a retrieval ceiling of 500 records per query; Block C was retrieved and screened in full (Table S2); Block D is a methodological control.

ID	Purpose	Query string	Records retrieved	Records screened	Screening basis
A1	Curcuminoid pharmacokinetics	("curcumin"[tiab] OR curcuminoid[tiab]) AND (pharmacokinetic[tiab] OR "bioavailability"[tiab] OR "tissue distribution"[tiab] OR "metabolism"[tiab]) AND [DATE]	5140	500	Scoping — compound-class pharmacokinetics (screened by title/abstract)
A2	Carotenoid (crocin/crocetin) pharmacokinetics	("crocin"[tiab] OR "crocetin"[tiab] OR "saffron"[tiab] OR "Crocus sativus"[tiab]) AND (pharmacokinetic[tiab] OR "bioavailability"[tiab] OR "tissue distribution"[tiab] OR "metabolism"[tiab]) AND [DATE]	281	281	Scoping — compound-class pharmacokinetics (screened by title/abstract)
A3	Flavonoid pharmacokinetics	("naringenin"[tiab] OR "quercetin"[tiab] OR "epigallocatechin gallate"[tiab] OR "EGCG"[tiab]) AND (pharmacokinetic[tiab] OR "bioavailability"[tiab] OR "tissue distribution"[tiab] OR "metabolism"[tiab]) AND [DATE]	6166	500	Scoping — compound-class pharmacokinetics (screened by title/abstract)
A4	Stilbene pharmacokinetics	("resveratrol"[tiab] OR stilbene[tiab] OR "pterostilbene"[tiab]) AND (pharmacokinetic[tiab] OR "bioavailability"[tiab] OR "tissue distribution"[tiab] OR "metabolism"[tiab]) AND [DATE]	4024	500	Scoping — compound-class pharmacokinetics (screened by title/abstract)
A5	Phytoestrogen pharmacokinetics / speciation	("daidzein"[tiab] OR "genistein"[tiab] OR "equol"[tiab] OR isoflavone[tiab]) AND (pharmacokinetic[tiab] OR "bioavailability"[tiab] OR "tissue distribution"[tiab] OR "metabolism"[tiab]) AND [DATE]	2813	500	Scoping — compound-class pharmacokinetics (screened by title/abstract)
B1	Endometrium x delivery systems	("endometrium"[tiab] OR "endometrial"[tiab]) AND ("drug delivery"[tiab] OR nanoparticle[tiab] OR nanocarrier[tiab] OR liposome[tiab] OR extracellular vesicle[tiab] OR exosome[tiab]) AND [DATE]	970	500	Scoping — target and delivery problem (screened by title/abstract)
B2	Receptivity/implantation window x formulation	("implantation window"[tiab] OR "window of implantation"[tiab] OR "endometrial receptivity"[tiab] OR "decidualization"[tiab] OR "decidualisation"[tiab]) AND ("drug delivery"[tiab] OR nanoparticle[tiab] OR "formulation"[tiab] OR "carrier"[tiab]) AND [DATE]	66	66	Scoping — target and delivery problem (screened by title/abstract)
B3	Vaginal route / first uterine pass	("vaginal"[tiab] AND ("drug delivery"[tiab] OR "first uterine pass"[tiab] OR "uterine first pass"[tiab])) AND [DATE]	771	500	Scoping — target and delivery problem (screened by title/abstract)

B4	Intrauterine delivery systems	("intrauterine"[tiab] AND (hydrogel[tiab] OR "drug delivery"[tiab] OR "sustained release"[tiab])) AND [DATE]	217	217	Scoping — target and delivery problem (screened by title/abstract)
B5	Preimplantation embryo x particles	(preimplantation embryo[tiab] OR blastocyst[tiab] OR "zona pellucida"[tiab]) AND (nanoparticle[tiab] OR lipid nanoparticle[tiab] OR nanoplastic[tiab] OR microplastic[tiab]) AND [DATE]	119	119	Scoping — target and delivery problem (screened by title/abstract)
B6	Human endometrial / embryo tissue models	(endometrial organoid[tiab] OR assembloid[tiab] OR blastoid[tiab] OR "endometrium-on-a-chip"[tiab] OR "endometrium on chip"[tiab]) AND [DATE]	1411	500	Scoping — target and delivery problem (screened by title/abstract)
C1	Curcuminoids x endometrium x concentration	("curcumin"[tiab] OR curcuminoid[tiab]) AND endometri[tiab] AND (concentration[tiab] OR tissue level[tiab] OR "tissue distribution"[tiab] OR pharmacokinetic[tiab]) AND [DATE]	13	13	Evidentiary — basis of the negative claim (screened in full)
C2	Carotenoids x endometrium x concentration	("crocin"[tiab] OR "crocetin"[tiab] OR "saffron"[tiab] OR "Crocus sativus"[tiab]) AND endometri[tiab] AND (concentration[tiab] OR tissue level[tiab] OR "tissue distribution"[tiab] OR pharmacokinetic[tiab]) AND [DATE]	0	0	Evidentiary — basis of the negative claim (screened in full)
C3	Flavonoids x endometrium x concentration	("naringenin"[tiab] OR "quercetin"[tiab] OR "epigallocatechin gallate"[tiab] OR "EGCG"[tiab]) AND endometri[tiab] AND (concentration[tiab] OR tissue level[tiab] OR "tissue distribution"[tiab] OR pharmacokinetic[tiab]) AND [DATE]	23	23	Evidentiary — basis of the negative claim (screened in full)
C4	Stilbenes x endometrium x concentration	("resveratrol"[tiab] OR stilbene[tiab] OR "pterostilbene"[tiab]) AND endometri[tiab] AND (concentration[tiab] OR tissue level[tiab] OR "tissue distribution"[tiab] OR pharmacokinetic[tiab]) AND [DATE]	18	18	Evidentiary — basis of the negative claim (screened in full)
C5	Phytoestrogens x endometrium x concentration	("daidzein"[tiab] OR "genistein"[tiab] OR "equol"[tiab] OR isoflavone[tiab]) AND endometri[tiab] AND (concentration[tiab] OR tissue level[tiab] OR "tissue distribution"[tiab] OR pharmacokinetic[tiab]) AND [DATE]	61	61	Evidentiary — basis of the negative claim (screened in full)
C6	Any of the five classes x explicit tissue-concentration phrase	(("curcumin"[tiab] OR curcuminoid[tiab]) OR ("crocin"[tiab] OR "crocetin"[tiab] OR "saffron"[tiab] OR "Crocus sativus"[tiab]) OR ("naringenin"[tiab] OR "quercetin"[tiab] OR "epigallocatechin gallate"[tiab] OR "EGCG"[tiab]) OR ("resveratrol"[tiab] OR stilbene[tiab] OR "pterostilbene"[tiab]) OR ("daidzein"[tiab] OR "genistein"[tiab] OR "equol"[tiab] OR isoflavone[tiab])) AND (endometrial concentration[tiab] OR endometrial tissue concentration[tiab] OR uterine tissue concentration[tiab] OR endometrial tissue level[tiab]) AND [DATE]	0	0	Evidentiary — basis of the negative claim (screened in full)
D1	CONTROL: steroids x endometrium x tissue concentration	("progesterone"[tiab] OR "estradiol"[tiab] OR "oestradiol"[tiab] OR "danazol"[tiab]) AND endometri[tiab] AND (tissue concentration[tiab] OR tissue level[tiab] OR endometrial concentration[tiab]) AND [DATE]	102	102	Methodological control — does the query form retrieve tissue-concentration studies?

Deduplicated union of Blocks A and B: 4,066 unique records.

Block C: 115 records retrieved across six queries; 105 unique after deduplication; all 105 abstracts retrieved and screened; 30 given a detailed sentence-by-sentence assessment (Table S2).

Block D (control): 102 records. The control retrieved the human endometrial tissue measurements cited in Section 3.3 (PMID 9194669, Bulletti et al. 1997; PMID 15346659, Ficicioglu et al. 2004; PMID 9329850, de Ziegler et al. 1997) and did **not** retrieve PMID 11108871 (Levy et al. 2000), consistent with that study reporting plasma pharmacokinetics only. The strategy therefore returns endometrial tissue-concentration studies when they exist.

Table S2. Screening record for the 105 unique Block C articles

Each record was assessed against one question: *does this record report a concentration of one of the five compound classes measured in reproductive tissue or in uterine fluid?* "Detailed assessment" indicates records for which the complete abstract was examined sentence by sentence against that question and the matrix of every reported concentration recorded. Records are listed newest first.

#	PMID	Year	Title	Outcome	Matrix in which a concentration was reported	Detailed assessment	Reason
1	41983360	2026	The Effect of Curcumin on Angiogenic and Proliferative Factors inHuman Endometriotic Cells	EXCLUDED	none	yes	In vitro cell-based study; the only concentrations reported are treatment concentrations applied to the culture medium.
2	41797792	2026	From bench to bedside: unlocking the anti-inflammatory, antioxidant, and anticancer promise of curcumin in gynecology	EXCLUDED	none	no	Review, guideline or advisory article (PubMed publication type); reports no primary measurement of a compound in reproductive tissue.
3	41587394	2026	Targeting Cancer Through Oxidative Stress: The Role of Epigallocatechin Gallate in Reactive Oxygen Species Modulation	EXCLUDED	none	no	Review, guideline or advisory article (PubMed publication type); reports no primary measurement of a compound in reproductive tissue.
4	41573293	2026	Endometrial Expression of Angiogenesis-related Factors Following Ovarian Stimulation and Epigallocatechin Gallate Administration	NEAR MISS	none	yes	In vivo murine study of EGCG with endometrial endpoints (VEGF protein, miR-16-5p, CD31 density); no EGCG measurement in any compartment.
5	41506495	2026	Dual effects of metformin and resveratrol on compromising viability of endometrial cancer cells	EXCLUDED	none	no	In vitro cell-based study; the only concentrations reported are treatment concentrations applied to the culture medium.
6	41373108	2025	Role of Phytoestrogens in Endometriosis	EXCLUDED	none	no	Review, guideline or advisory article (PubMed publication type); reports no primary measurement of a compound in reproductive tissue.
7	41279406	2025	The novel curcumin analog AKT-100 targets mutant p53	EXCLUDED	none	no	Preprint, not peer reviewed. No concentration of a study

			in gynecologic cancer cells				compound was measured in reproductive tissue or uterine fluid.
8	39705870	2025	Therapeutic potential of adult stem cells-derived mitochondria transfer combined with curcumin administration into ARPE-19 cells in age-related macular degeneration model	EXCLUDED	none	no	In vitro cell-based study; the only concentrations reported are treatment concentrations applied to the culture medium.
9	40098247	2025	Nanohydrogel of Curcumin/Berberine Co-Crystals Induces Apoptosis via Dual Covalent/Noncovalent Inhibition of Caspases in Endometrial Cancer Cell Lines: The Synergy Between Pharmacokinetics and Pharmacodynamics	EXCLUDED	none	no	In vitro cell-based study; the only concentrations reported are treatment concentrations applied to the culture medium.
10	41669294	2025	Serum Reproductive Hormone Profiles and Endometrial Expression of miR-16-5p, VEGF Gene, and Protein, and CD31-Positive Cells following Progesterone and EGCG Administration	EXCLUDED	serum	yes	In vivo study with reproductive, hormonal or systemic endpoints; no concentration of a study compound measured in reproductive tissue.
11	41201718	2025	Quercetin suppresses endometrial cancer stem cells via ERα-mediated inhibition of STAT3 signaling	EXCLUDED	serum	no	In vitro cell-based study; the only concentrations reported are treatment concentrations applied to the culture medium.
12	39861342	2025	Role of Epigallocatechin Gallate in Selected Malignant Neoplasms in Women	EXCLUDED	plasma	no	Review, guideline or advisory article (PubMed publication type); reports no primary measurement of a compound in reproductive tissue.
13	40411697	2025	Pterostilbene as a Multifaceted Anticancer Agent: Molecular Mechanisms, Therapeutic Potential and Future Directions	EXCLUDED	none	no	Review, guideline or advisory article (PubMed publication type); reports no primary measurement of a compound in reproductive tissue.
14	38948281	2024	[Quercetin Alleviates H(2)O(2)-Induced Oxidative Stress Damage to Human Endometrial Stromal Cells by Inhibiting the p38 MAPK/NOX4 Signaling Pathway]	EXCLUDED	none	no	In vivo study with reproductive, hormonal or systemic endpoints; no concentration of a study compound measured in reproductive tissue.
15	38279186	2024	Mechanisms of Bushen Tiaoxue Granules against controlled ovarian hyperstimulation-induced abnormal morphology of	EXCLUDED	none	no	No concentration of a study compound was measured in reproductive tissue or uterine fluid.

			endometrium based on network pharmacology				
16	38862934	2024	Resveratrol activates MAPK/ERK pathway to regulate oestrogen metabolism in type I endometrial cancer	EXCLUDED	serum	no	Pharmacokinetic or analytical study quantifying a compound in serum; no measurement in reproductive tissue or uterine fluid.
17	37847468	2024	Estrogen receptor α interaction of zearalenone and its phase I metabolite α-zearalenol in combination with soy isoflavones in hERα-HeLa-9903 cells	EXCLUDED	none	no	In vitro cell-based study; the only concentrations reported are treatment concentrations applied to the culture medium.
18	35641803	2023	Curcumin-loaded human endometrial stem cells derived exosomes as an effective carrier to suppress alpha-synuclein aggregates in 6OHDA-induced Parkinson's disease mouse model	EXCLUDED	none	no	No concentration of a study compound was measured in reproductive tissue or uterine fluid.
19	37107282	2023	The Importance of Natural Antioxidants in Female Reproduction	EXCLUDED	none	no	Review, guideline or advisory article (PubMed publication type); reports no primary measurement of a compound in reproductive tissue.
20	36824010	2023	Resveratrol improves ovarian state by inhibiting apoptosis of granulosa cells	EXCLUDED	none	no	Human clinical or observational study with clinical, hormonal or histological endpoints; no compound concentration measured in reproductive tissue.
21	35041229	2022	An overview of the therapeutic effects of curcumin in reproductive disorders with a focus on the antiinflammatory and immunomodulatory activities	EXCLUDED	none	no	Review, guideline or advisory article (PubMed publication type); reports no primary measurement of a compound in reproductive tissue.
22	36304154	2022	Determination of (-)-epigallocatechin-3-gallate octaacetate and its metabolites in plasma of rats for pharmacokinetic study by ultra-performance-liquid-chromatography coupled to quadrupole-time-of-flight-mass-spectrometry	EXCLUDED	plasma	yes	Pharmacokinetic or analytical study quantifying a compound in plasma; no measurement in reproductive tissue or uterine fluid.
23	35956866	2022	Resveratrol Effects on the Reproductive System in Ovariectomized Rats: Deciphering Possible Mechanisms	EXCLUDED	none	yes	In vivo study with reproductive, hormonal or systemic endpoints; no concentration of a study compound measured in

							reproductive tissue.
24	35593501	2022	Effects of isoflavone supplementation on endometrial thickness, endometrial hyperplasia, and cancer in ovariectomized cats	EXCLUDED	none	yes	In vivo study with reproductive, hormonal or systemic endpoints; no concentration of a study compound measured in reproductive tissue.
25	35784757	2022	An Efficient UPLC-MS/MS Method Established to Detect Relugolix Concentration in Rat Plasma	EXCLUDED	plasma	yes	Pharmacokinetic or analytical study quantifying a compound in plasma; no measurement in reproductive tissue or uterine fluid.
26	34801523	2022	Developmental exposure to phytoestrogens found in soy: New findings and clinical implications	EXCLUDED	serum	no	Review, guideline or advisory article (PubMed publication type); reports no primary measurement of a compound in reproductive tissue.
27	34212031	2021	Anti-Inflammatory Activity and Quality Control of Erysimum cheiri (L.) Crantz	EXCLUDED	none	no	In vitro cell-based study; the only concentrations reported are treatment concentrations applied to the culture medium.
28	33061289	2020	Quercetin Inhibits Adenomyosis by Attenuating Cell Proliferation, Migration and Invasion of Ectopic Endometrial Stromal Cells	EXCLUDED	none	yes	Human clinical or observational study with clinical, hormonal or histological endpoints; no compound concentration measured in reproductive tissue.
29	32181408	2020	Inhibitory effect of chrysin on estrogen biosynthesis by suppression of enzyme aromatase (CYP19): A systematic review	EXCLUDED	none	no	Review, guideline or advisory article (PubMed publication type); reports no primary measurement of a compound in reproductive tissue.
30	32896495	2020	Inhibitory effect of resveratrol on the growth and angiogenesis of human endometrial tissue in an In Vitro three-dimensional model of endometriosis	EXCLUDED	none	yes	In vitro cell-based study; the only concentrations reported are treatment concentrations applied to the culture medium.
31	30259980	2019	Curcumin attenuates proangiogenic and proinflammatory factors in human eutopic endometrial stromal cells through the NF-κB signaling pathway	EXCLUDED	none	no	No concentration of a study compound was measured in reproductive tissue or uterine fluid.
32	31223026	2019	Synergistic effects of quercetin and selenium on oxidative stress in endometrial adenocarcinoma cells	EXCLUDED	none	yes	In vitro cell-based study; the only concentrations reported are treatment concentrations applied to the culture medium.
33	30777471	2019	The modulating effects of Resveratrol on the	NEAR MISS	endometrial tissue and fluid sampled, compound not	yes	Randomized trial of oral resveratrol 400 mg for 12-14

			expression of MMP-2 and MMP-9 in endometriosis women: a randomized exploratory trial		assayed		weeks; endometrial tissue, endometrial fluid and blood sampled before and after. The analytes measured were MMP-2 and MMP-9; resveratrol itself was not quantified in any compartment.
34	30070342	2018	Effects of curcumin on the role of MMP-2 in endometrial cancer cell proliferation and invasion	EXCLUDED	none	yes	In vivo study with reproductive, hormonal or systemic endpoints; no concentration of a study compound measured in reproductive tissue.
35	30283786	2018	The Hop Polyphenols Xanthohumol and 8-Prenyl-Naringenin Antagonize the Estrogenic Effects of Fusarium Mycotoxins in Human Endometrial Cancer Cells	EXCLUDED	none	no	In vitro cell-based study; the only concentrations reported are treatment concentrations applied to the culture medium.
36	29636868	2018	Novasoy and genistein inhibit endometrial cancer cell proliferation through disruption of the AKT/mTOR and MAPK signaling pathways	EXCLUDED	none	no	In vitro cell-based study; the only concentrations reported are treatment concentrations applied to the culture medium.
37	30090542	2017	Modulation of estrogen synthesis and metabolism by phytoestrogens in vitro and the implications for women's health	EXCLUDED	none	no	Review, guideline or advisory article (PubMed publication type); reports no primary measurement of a compound in reproductive tissue.
38	28229914	2017	Quercetin exerts preventive, ameliorative and prophylactic effects on cadmium chloride - induced oxidative stress in the uterus and ovaries of female Wistar rats	EXCLUDED	serum	yes	No concentration of a study compound was measured in reproductive tissue or uterine fluid.
39	28415020	2017	Simultaneous determination of ten bioactive constituents of Sanjie Zhentong Capsule in rat plasma by ultra-high-performance liquid chromatography tandem mass spectrometry and its application to a pharmacokinetic study	EXCLUDED	plasma	yes	Pharmacokinetic or analytical study quantifying a compound in plasma; no measurement in reproductive tissue or uterine fluid.
40	29241917	2017	Pueraria mirifica inhibits 17β-estradiol-induced cell proliferation of human endometrial mesenchymal stem cells	EXCLUDED	none	no	No concentration of a study compound was measured in reproductive tissue or uterine fluid.
41	28596011	2017	Emmenagogue properties of Milicia excelsa (Welw.) C.C.	EXCLUDED	serum	no	In vivo study with reproductive, hormonal or

			Berg (Moraceae) based, at least in part, on its ability to correlate the activity of the hypothalamic-pituitary axis to that of the ovaries				systemic endpoints; no concentration of a study compound measured in reproductive tissue.
42	27739238	2017	Combinatory estrogenic effects between the isoflavone genistein and the mycotoxins zearalenone and alternariol in vitro	EXCLUDED	none	no	In vitro cell-based study; the only concentrations reported are treatment concentrations applied to the culture medium.
43	28031375	2017	Urinary Phytoestrogen Concentrations Are Not Associated with Incident Endometriosis in Premenopausal Women	NEAR MISS	urine	yes	Reports urinary phytoestrogen concentrations in relation to endometriosis; urine is not reproductive tissue.
44	29949703	2016	Effect of quercetin on the expression of Bcl-2/Bax apoptotic proteins in endometrial cells of lipopolysaccharide-induced-abortion	EXCLUDED	none	yes	In vivo study with reproductive, hormonal or systemic endpoints; no concentration of a study compound measured in reproductive tissue.
45	27817194	2016	Suppression of isoproterenol-induced apoptosis in H9c2 cardiomyoblast cells by daidzein through activation of Akt	EXCLUDED	serum	no	In vitro cell-based study; the only concentrations reported are treatment concentrations applied to the culture medium.
46	26817207	2015	Isoflavone Genistein Modulates the Protein Expression of Toll-like Receptors in Cancerous Human Endometrial Cells	EXCLUDED	none	no	In vitro cell-based study; the only concentrations reported are treatment concentrations applied to the culture medium.
47	25661255	2015	Soy isoflavones improves endometrial barrier through tight junction gene expression	EXCLUDED	none	no	In vitro cell-based study; the only concentrations reported are treatment concentrations applied to the culture medium.
48	25003624	2015	Soy Isoflavones for Reducing Bone Loss Study: effects of a 3-year trial on hormones, adverse events, and endometrial thickness in postmenopausal women	EXCLUDED	plasma, urine	yes	Clinical trial with clinical, hormonal or histological endpoints; no compound concentration measured in reproductive tissue.
49	25556280	2014	Curcumin promotes the apoptosis of human endometrial carcinoma cells by downregulating the expression of androgen receptor through Wnt signal pathway	EXCLUDED	none	no	In vitro cell-based study; the only concentrations reported are treatment concentrations applied to the culture medium.
50	24604232	2014	Resveratrol and endometrium: a closer look at an active ingredient of red wine using in vivo and in vitro models	NEAR MISS	none	yes	Human endometrial xenograft after resveratrol pellets of defined mass, plus an Ishikawa estrogenicity assay; receptor and proliferation endpoints only,

							with no tissue assay of resveratrol.
51	24073920	2014	Long-term resveratrol treatment prevents ovariectomy-induced osteopenia in rats without hyperplastic effects on the uterus	EXCLUDED	serum, urine	yes	In vivo study with reproductive, hormonal or systemic endpoints; no concentration of a study compound measured in reproductive tissue.
52	23305490	2013	A role for two-pore potassium (K2P) channels in endometrial epithelial function	EXCLUDED	none	no	In vitro cell-based study; the only concentrations reported are treatment concentrations applied to the culture medium.
53	23771522	2013	Dietary soy effects on mammary gland development during the pubertal transition in nonhuman primates	EXCLUDED	none	no	No concentration of a study compound was measured in reproductive tissue or uterine fluid.
54	23569430	2013	Isoflavone genistein induces fluid secretion and morphological changes in the uteri of post-pubertal rats	EXCLUDED	none	yes	In vivo study with reproductive, hormonal or systemic endpoints; no concentration of a study compound measured in reproductive tissue.
55	21934634	2012	The effects of natural S-equol supplementation on skin aging in postmenopausal women: a pilot randomized placebo-controlled trial	EXCLUDED	serum	no	In vivo study with reproductive, hormonal or systemic endpoints; no concentration of a study compound measured in reproductive tissue.
56	20844278	2011	Resveratrol inhibits development of experimental endometriosis in vivo and reduces endometrial stromal cell invasiveness in vitro	EXCLUDED	none	yes	In vivo study with reproductive, hormonal or systemic endpoints; no concentration of a study compound measured in reproductive tissue.
57	21685820	2011	The role of soy isoflavones in menopausal health: report of The North American Menopause Society/Wulf H. Utian Translational Science Symposium in Chicago, IL (October 2010)	EXCLUDED	none	no	Human clinical or observational study with clinical, hormonal or histological endpoints; no compound concentration measured in reproductive tissue.
58	21281625	2011	Genistein effects on stromal cells determines epithelial proliferation in endometrial co-cultures	EXCLUDED	none	yes	In vitro cell-based study; the only concentrations reported are treatment concentrations applied to the culture medium.
59	21177797	2011	Clinical outcomes of a 2-y soy isoflavone supplementation in menopausal women	EXCLUDED	serum	yes	Clinical trial with clinical, hormonal or histological endpoints; no compound concentration measured in reproductive tissue.
60	20457218	2010	Beta-arrestin 2 modulates resveratrol-induced apoptosis and regulation of	EXCLUDED	none	no	In vitro cell-based study; the only concentrations reported are treatment concentrations

			Akt/GSK3ß pathways				applied to the culture medium.
61	19321194	2009	Regulation of Vascular Endothelial Growth Factor in endometrial tumour cells by resveratrol and EGCG	EXCLUDED	none	no	In vitro cell-based study; the only concentrations reported are treatment concentrations applied to the culture medium.
62	19007764	2009	AKR1C3 as a potential target for the inhibitory effect of dietary flavonoids	EXCLUDED	none	no	No concentration of a study compound was measured in reproductive tissue or uterine fluid.
63	19250196	2009	Estrogen sulfotransferases in breast and endometrial cancers	EXCLUDED	none	no	Review, guideline or advisory article (PubMed publication type); reports no primary measurement of a compound in reproductive tissue.
64	19686426	2009	Genistein supplements might not induce clitoral modifications in postmenopausal women: a prospective, pilot study	EXCLUDED	plasma	no	Clinical trial with clinical, hormonal or histological endpoints; no compound concentration measured in reproductive tissue.
65	19322873	2009	Estrogen/isoflavone interactions in cynomolgus macaques (Macaca fascicularis)	EXCLUDED	none	yes	Review, guideline or advisory article (PubMed publication type); reports no primary measurement of a compound in reproductive tissue.
66	19549922	2009	Stimulating the GPR30 estrogen receptor with a novel tamoxifen analog activates SF-1 and promotes endometrial cell proliferation	EXCLUDED	none	no	In vitro cell-based study; the only concentrations reported are treatment concentrations applied to the culture medium.
67	18980187	2008	Development of a stable dual cell-line GFP expression system to study estrogenic endocrine disruptors	EXCLUDED	none	no	In vitro cell-based study; the only concentrations reported are treatment concentrations applied to the culture medium.
68	19197458	2008	[Treatment with isoflavones replaces estradiol effect on the tissue fat accumulation from ovariectomized rats]	EXCLUDED	none	no	In vivo study with reproductive, hormonal or systemic endpoints; no concentration of a study compound measured in reproductive tissue.
69	18843000	2008	Past oral contraceptive use and current dietary soy isoflavones influence estrogen metabolism in postmenopausal monkeys (Macaca fascicularis)	EXCLUDED	urine	yes	In vivo study with reproductive, hormonal or systemic endpoints; no concentration of a study compound measured in reproductive tissue.
70	18437911	2008	Genistein inhibits proliferation of human endometrial endothelial cell in vitro	EXCLUDED	none	no	In vitro cell-based study; the only concentrations reported are treatment concentrations applied to the culture medium.

71	18047477	2008	Effects of lactational exposure to soy isoflavones on reproductive system in neonatal female rats	EXCLUDED	serum	yes	In vivo study with reproductive, hormonal or systemic endpoints; no concentration of a study compound measured in reproductive tissue.
72	18252965	2008	Isoflavones regulate secretion of leukemia inhibitory factor and transforming growth factor {beta} and expression of glycodelin in human endometrial epithelial cells	EXCLUDED	plasma	no	In vitro cell-based study; the only concentrations reported are treatment concentrations applied to the culture medium.
73	17343919	2007	Antioxidant activity of N-acetylcysteine, flavonoids and alpha-tocopherol on endometrial cells in culture	EXCLUDED	none	no	In vitro cell-based study; the only concentrations reported are treatment concentrations applied to the culture medium.
74	17051425	2007	Lycopene and other carotenoids inhibit estrogenic activity of 17beta-estradiol and genistein in cancer cells	EXCLUDED	none	no	In vitro cell-based study; the only concentrations reported are treatment concentrations applied to the culture medium.
75	16566672	2006	The chemical and biologic profile of a red clover (Trifolium pratense L.) phase II clinical extract	EXCLUDED	none	no	In vitro cell-based study; the only concentrations reported are treatment concentrations applied to the culture medium.
76	16963825	2006	Phyto- and endogenous estrogens differently activate intracellular calcium ion mobilization in bovine endometrial cells	EXCLUDED	none	no	In vitro cell-based study; the only concentrations reported are treatment concentrations applied to the culture medium.
77	16705744	2006	Changes in gene expressions elicited by physiological concentrations of genistein on human endometrial cancer cells	EXCLUDED	none	yes	In vitro cell-based study; the only concentrations reported are treatment concentrations applied to the culture medium.
78	16598814	2006	Stimulation of estradiol glucuronidation: a protective mechanism against estradiol-mediated carcinogenesis?	EXCLUDED	none	no	No concentration of a study compound was measured in reproductive tissue or uterine fluid.
79	16534752	2006	Estrogens modulate the gene expression of Wnt-7a in cultured endometrial adenocarcinoma cells	EXCLUDED	none	no	In vitro cell-based study; the only concentrations reported are treatment concentrations applied to the culture medium.
80	16478248	2006	Seasonal variation of red clover (Trifolium pratense L., Fabaceae) isoflavones and estrogenic activity	EXCLUDED	none	no	In vitro cell-based study; the only concentrations reported are treatment concentrations applied to the culture medium.
81	16418439	2006	Soy protein, isoflavones, and cardiovascular health: an American Heart Association	EXCLUDED	none	no	Review, guideline or advisory article (PubMed publication type); reports no

			Science Advisory for professionals from the Nutrition Committee				primary measurement of a compound in reproductive tissue.
82	16967098	2006	The influence of polychlorinated biphenyls (PCBs) and phytoestrogens in vitro on functioning of reproductive tract in cow	EXCLUDED	none	no	Review, guideline or advisory article (PubMed publication type); reports no primary measurement of a compound in reproductive tissue.
83	15901641	2005	Stimulation of macrophage migration inhibitory factor expression in endometrial stromal cells by interleukin 1, beta involving the nuclear transcription factor NFkappaB	EXCLUDED	peritoneal fluid	no	In vitro cell-based study; the only concentrations reported are treatment concentrations applied to the culture medium.
84	16081271	2005	Uterine phenotype of young adult rats exposed to dietary soy or genistein during development	PARTIAL POSITIVE	uterine tissue (rat)	yes	Reports uterine tissue and serum concentrations of total genistein in rats after lifetime dietary exposure. Whole uterus rather than endometrium; rat rather than human; total rather than speciated; lifetime dietary exposure rather than a defined dose.
85	15993745	2005	Estrogenic and genotoxic potential of equol and two hydroxylated metabolites of Daidzein in cultured human Ishikawa cells	EXCLUDED	none	no	In vitro cell-based study; the only concentrations reported are treatment concentrations applied to the culture medium.
86	15968319	2005	[Regulation of genistein on the levels of ERalpha, ERbeta mRNA in uterine endometrial cancer cells]	EXCLUDED	none	yes	In vitro cell-based study; the only concentrations reported are treatment concentrations applied to the culture medium.
87	15734722	2005	Soybean-derived phytoestrogens regulate prostaglandin secretion in endometrium during cattle estrous cycle and early pregnancy	EXCLUDED	serum, urine	no	In vivo bovine study: daidzein and genistein in fodder and their metabolites equol and p-ethyl-phenol quantified in serum and urine, with endometrial prostaglandin endpoints. The compounds were measured, but in serum and urine rather than in reproductive tissue.
88	14713553	2004	Effects of dietary isoflavone aglycones on the reproductive tract of male and female mice	EXCLUDED	plasma	no	In vivo study with reproductive, hormonal or systemic endpoints; no concentration of a study compound measured in reproductive tissue.
89	13679094	2003	High isoflavone content and estrogenic activity of 25 year-old Glycine max tissue	EXCLUDED	none	no	In vitro cell-based study; the only concentrations reported are treatment concentrations

			cultures				applied to the culture medium.
90	12840226	2003	Indole-3-carbinol is a negative regulator of estrogen	EXCLUDED	none	no	Review, guideline or advisory article (PubMed publication type); reports no primary measurement of a compound in reproductive tissue.
91	12820358	2003	Immunohistochemical expression of steroid receptors and glycodelin A in isolated proliferative human endometrial glandular cells after stimulation with tamoxifen and phytoestrogens (genistein and daidzein)	EXCLUDED	none	no	Human clinical or observational study with clinical, hormonal or histological endpoints; no compound concentration measured in reproductive tissue.
92	12650720	2002	Stimulation of alkaline phosphatase activity in Ishikawa cells induced by various phytoestrogens and synthetic estrogens	EXCLUDED	none	no	In vitro cell-based study; the only concentrations reported are treatment concentrations applied to the culture medium.
93	12466350	2002	Estrogenicity of isoflavones on human endometrial stromal and glandular cells	EXCLUDED	none	no	In vitro cell-based study; the only concentrations reported are treatment concentrations applied to the culture medium.
94	12370124	2002	Responsiveness of endometrial genes Connexin26, Connexin43, C3 and clusterin to primary estrogen, selective estrogen receptor modulators, phyto- and xenoestrogens	EXCLUDED	plasma	no	No concentration of a study compound was measured in reproductive tissue or uterine fluid.
95	11880595	2002	Hormonal effects of soy in premenopausal women and men	EXCLUDED	plasma, serum, urine	no	Review, guideline or advisory article (PubMed publication type); reports no primary measurement of a compound in reproductive tissue.
96	11507064	2001	Resveratrol exhibits cytostatic and antiestrogenic properties with human endometrial adenocarcinoma (Ishikawa) cells	EXCLUDED	none	no	In vitro cell-based study; the only concentrations reported are treatment concentrations applied to the culture medium.
97	11294976	2001	Pharmacologic, but not dietary, genistein supports endometriosis in a rat model	EXCLUDED	none	yes	In vivo study with reproductive, hormonal or systemic endpoints; no concentration of a study compound measured in reproductive tissue.
98	11075748	2000	Soy isoflavones: are they useful in menopause?	EXCLUDED	serum	no	Review, guideline or advisory article (PubMed publication type); reports no primary measurement of a

							compound in reproductive tissue.
99	10973655	2000	Exposure of human endometrium to environmental estrogens, antiandrogens, and organochlorine compounds	EXCLUDED	none	no	Human clinical or observational study with clinical, hormonal or histological endpoints; no compound concentration measured in reproductive tissue.
100	10656875	1999	Tyrosine phosphorylation of focal adhesion kinase and paxillin regulates the signaling mechanism of the rapid nongenomic action of dexamethasone on actin cytoskeleton	EXCLUDED	none	no	In vitro cell-based study; the only concentrations reported are treatment concentrations applied to the culture medium.
101	10330684	1999	Development of a stably transfected estrogen receptor-mediated luciferase reporter gene assay in the human T47D breast cancer cell line	EXCLUDED	none	no	In vitro cell-based study; the only concentrations reported are treatment concentrations applied to the culture medium.
102	9920082	1999	Soy isoflavones exert modest hormonal effects in premenopausal women	EXCLUDED	plasma	no	Clinical trial with clinical, hormonal or histological endpoints; no compound concentration measured in reproductive tissue.
103	11907938	1998	Phytoestrogens and the menopause	EXCLUDED	urine	no	Review, guideline or advisory article (PubMed publication type); reports no primary measurement of a compound in reproductive tissue.
104	9721258	1998	Characterization of estrogenicity of phytoestrogens in an endometrial-derived experimental model	EXCLUDED	none	no	In vitro cell-based study; the only concentrations reported are treatment concentrations applied to the culture medium.
105	8641611	1996	Platelet-activating factor enhances production of insulin-like growth factor binding proteins in a human adenocarcinoma cell line (HEC-1A)	EXCLUDED	none	no	In vitro cell-based study; the only concentrations reported are treatment concentrations applied to the culture medium.

Screening limitation. Screening was performed on complete abstracts rather than on full texts. A study that measured a compound concentration in reproductive tissue would be expected to state so in its abstract, which is the basis for this procedure; the residual risk is that a tissue concentration reported only in a results table of a paper whose abstract does not mention it would be missed. This is stated as a limitation rather than excluded.

Summary. Of 105 unique records, 1 reported a measured tissue concentration of a study compound (PMID 16081271; total genistein in whole rat uterus after lifetime dietary exposure), 4 were near misses in which the relevant compartment was sampled or the compound was administered in vivo without being assayed, and 100 reported no concentration of a study compound in reproductive tissue or uterine fluid. **No record reported an endometrial tissue or uterine fluid concentration of any of the five compound classes in women.**
